# Mixed methods study on latent tuberculosis among agate stone workers and advocacy for testing silica dust exposed individuals in India

**DOI:** 10.1038/s41598-024-64837-4

**Published:** 2024-06-15

**Authors:** Mihir P. Rupani, Rakesh Balachandar, Gitika Kharkwal, Nikhil P. Kulkarni, Bhavesh V. Modi, Rutu N. Asodia, Krishna K. Vaghela, Deizy R. Nimavat

**Affiliations:** 1 Division of Health Sciences, ICMR - National Institute of Occupational Health (NIOH), Indian Council of Medical Research (ICMR), Meghaninagar, Ahmedabad, Gujarat 380016 India; 2 Division of Biological Sciences, ICMR - National Institute of Occupational Health (NIOH), Indian Council of Medical Research (ICMR), Meghaninagar, Ahmedabad, Gujarat 380016 India; 3 Division of Chemical Sciences, ICMR - National Institute of Occupational Health (NIOH), Indian Council of Medical Research (ICMR), Meghaninagar, Ahmedabad, Gujarat 380016 India; 4 Department of Community and Family Medicine, All India Institute of Medical Sciences (AIIMS), Rajkot, Gujarat 360006 India

**Keywords:** Latent tuberculosis infection, Silicosis, Agate workers, Key population, High risk, Occupational settings, Dust exposure duration, Direct-treat strategy, Tuberculosis preventive treatment, India, Tuberculosis, Disease prevention, Occupational health, Public health

## Abstract

The 2021 tuberculosis (TB) preventive treatment guidelines in India included silicosis as a screening group, yet latent TB infection (LTBI) testing for silica-dust-exposed individuals is underemphasized. Focusing on an estimated 52 million silica-dust-exposed workers, particularly agate-stone workers in Khambhat, Gujarat, our study aims to estimate LTBI prevalence, identify predictors, and gather insights from TB and silicosis experts. Employing a sequential explanatory mixed-methods approach, a cross-sectional study involved 463 agate-stone workers aged ≥ 20 years in Khambhat, using IGRA kits for LTBI testing. In-depth interviews with experts complemented quantitative findings. Among agate-stone workers, 58% tested positive for LTBI, with predictors including longer exposure, type of work, and BCG vaccination. Our findings reveal a nearly double burden of LTBI compared to the general population, particularly in occupations with higher silica dust exposure. Experts advocate for including silica-dust-exposed individuals in high-risk groups for LTBI testing, exploring cost-effective alternatives like improved skin sensitivity tests, and shorter TB preventive treatment regimens to enhance compliance. Future research should explore upfront TB preventive treatment for silica-dust-exposed individuals with high LTBI prevalence and optimal exposure duration. This study underscores the urgent need for policy changes and innovative approaches to TB prevention among silica-dust-exposed populations, impacting global occupational health strategies.

## Introduction

Latent tuberculosis infection (LTBI) denotes a persistent immune response to Mycobacterium tuberculosis antigens without active clinical TB^1^. Globally, almost 1.7 billion individuals, comprising nearly a quarter of the world population, harbor latent TB bacilli^2^. India, striving for TB elimination by 2025, grapples with an alarming 0.35–0.4 billion TB infections and 2.6 million annual TB cases^3–7^. Studies indicate a 5–10% progression from LTBI to active TB disease, typically within 2 years post-infection^8,9^.

Existing literature predominantly addresses occupational latent TB infection risks among healthcare personnel, neglecting industrial dust-exposed populations^10^. International studies, particularly in Germany, South Africa, China, and Sri Lanka, have reported varying prevalence rates of latent tuberculosis infection (LTBI) among dust-exposed populations, ranging from 36 to 89%^11–16^. In India, LTBI prevalence is 36% in the general population and rises to 41% in high-risk groups^17,18^. Notably, there is a lack of research examining LTBI prevalence specifically among dust-exposed populations within the Indian context^17,18^.

India’s 2021 guidelines for TB preventive treatment encompass silicosis as a screening group^5^. Despite recognizing elevated TB risk from silica dust^19–21^, national guidelines lack emphasis on LTBI testing for silica-dust-exposed individuals^5^. Acknowledging underreporting challenges in silicosis notifications, we extend our focus to an estimated 52 million silica-dust-exposed workers in India^22^. Within this demographic, agate-stone workers, particularly vulnerable to silica-dust, constitute our study cohort^23^. The study’s objectives encompass estimating LTBI prevalence, identifying predictors among agate-stone workers, and soliciting insights from TB and silicosis experts on this critical issue.

## Methods

### Study design and duration

We employed a sequential explanatory mixed-methods approach to investigate latent tuberculosis infection (LTBI) among agate-stone workers in Khambhat, Gujarat, located in western India. The choice of a sequential explanatory mixed-methods design was driven by the need to triangulate quantitative findings with qualitative insights, providing a comprehensive understanding. Our study utilized a cross-sectional design to quantify LTBI prevalence and identify predictors within the agate-stone worker population. Subsequently, we conducted in-depth interviews with experts in the fields of tuberculosis (TB) and silicosis to gain insights into the necessity of including silica-dust-exposed individuals as a crucial population for LTBI testing. We adopted a pragmatic approach in our study, focusing on the immediate and practical goal of enhancing LTBI and TB preventive treatment (TPT) program for agate-stone workers in Khambhat. Rather than being driven by a theoretical framework, our study was designed to directly address the unique challenges faced by this population. The qualitative aspect of our study adhered to the constructivist paradigm, a framework positing that individuals construct their perceptions of the world through experiences and interactions. Embracing this paradigm acknowledges that knowledge is dynamic and continuously reconstructed as people engage with diverse experiences and perspectives^24^. This approach aimed to provide a comprehensive understanding of study participants' subjective experiences and perspectives. Employing a descriptive design, we categorized and described the codes derived from the in-depth interviews. The cross-sectional study was executed from June to October 2023, followed by the in-depth interviews conducted between November 2023 and January 2024.

### Study setting

Our study was conducted in Khambhat, renowned for its agate-stone craftsmanship, situated in the Gujarat state, western India. Khambhat was strategically selected as our study setting due to its prominence in agate-stone work, offering a concentrated population exposed to silica dust. This setting provides a pertinent backdrop for investigating latent TB infection among agate-stone workers. This location hosts approximately 50,000 workers engaged in the cottage industry^23,25,26^. The primary activities among agate-stone workers include polishing, chipping, and drilling^27,28^. Notably, the water-drip method is extensively employed in polishing and drilling, where water plays a pivotal role in these processes^22,28^. Agate stones, constituting over 60% free silica, serve as the fundamental material for crafting jewellery and various decorative articles^26^. The silica dust concentrations observed during these processes (0.11–0.12 mg/m^3^) substantially exceed international standards, ranging from 0.025 to 0.05 mg/m^3^^29^. This elevated exposure underscores the significance of investigating latent tuberculosis infection (LTBI) among this population. Khambhat's agate-stone workers have been reported to endure a considerable burden of silicosis, with prevalence rates ranging from 18 to 69% across different studies^25,29^.

### Study population and sample size

#### Quantitative

The cross-sectional study included individuals aged ≥ 20 years, each with at least 5 years of experience in the agate-stone industry, operating primarily from their homes, and who willingly provided written informed consent^30,31^. Our inclusion criteria, targeting individuals aged ≥ 20 years, align with India's guidelines on TB preventive treatment defining adults in this age group, while the stipulation of ≥ 5 years of employment in the agate-stone industry is driven by the heightened risk of silica exposure among this occupational cohort^30,31^. Exclusion criteria comprised household contacts of active TB patients, presumptive pulmonary TB cases, individuals undergoing tuberculosis treatment, those with known allergies to TB drugs, diagnosed HIV-positive, pregnant, and engaged in current alcohol consumption. Sample size determination, conducted using EpiInfo software version 7 (https://www.cdc.gov/epiinfo/support/downloads.html),^32^ assumed a 47% latent TB infection prevalence based on a prior Chinese study^11^. With a 95% confidence level and 5% precision, the calculated sample size was 383, adjusted to 459 to accommodate a 20% loss to follow-up.

#### Qualitative

Conducting in-depth interviews, we engaged with experts in tuberculosis (TB), alongside district and state-level officials actively participating in the TB preventive treatment (TPT) program for household contacts. Our interviews also involved TB health workers and experts specializing in silicosis. The selection of participants was purposeful, targeting individuals possessing specific technical insights into latent TB infection and TB preventive treatment. This approach ensured diverse perspectives and enriched the qualitative insights necessary for a comprehensive understanding of the research questions. During these insightful conversations, crucial points were meticulously documented. The interview process continued until data saturation was achieved, meaning subsequent interviews yielded redundant information. Saturation verification was methodically carried out by comparing notes after each interview. This approach aligns with qualitative research standards, ensuring a thorough exploration of participants' perspectives and insights until thematic saturation was reached. In total, thirteen in-depth interviews were conducted, ensuring a comprehensive exploration of perspectives. Among those interviewed were four officials actively engaged in the TPT program, three TB health workers based in Khambhat, two state-level officials, two medical officers, one expert specializing in silicosis, and one expert well-versed in the TB care cascade. Importantly, every expert willingly contributed to the study, with none declining participation. Additionally, no repeat interviews were conducted.

### Data collection

#### Quantitative

To ascertain a representative cohort, a survey targeting agate-stone workers in Khambhat was meticulously conducted. Collaborating with the Khambhat health officer, areas exhibiting the highest concentrations of agate-stone workers—Shakkarpur, Akbarpur, Macchipur, Laldarwaja, and Nagara—were identified. Notably, Shakkarpur, Akbarpur, and Laldarwaja housed a significant proportion of polishers, while Macchipur predominantly accommodated chippers, and Nagara was the primary site for drilling activities in Khambhat. Systematically visiting households in these identified areas, our team gathered information on the occupation of residents and assessed their eligibility for participation. The project staff diligently enrolled eligible participants, administering a comprehensive written informed consent procedure. Subsequently, participants responded to a structured questionnaire prepared by the authors, covering sociodemographic details, occupational history, and relevant predictor variables (see Additional file 1). To mitigate potential bias during the survey, rigorous training was provided to the project staff responsible for participant enrollment. Clear instructions were given to ensure consistency in administering the written informed consent procedure and data collection, minimizing potential variations in responses. Blood samples, crucial for diagnosing latent TB infection, were collected and meticulously transported to our institute while maintaining cold chain. We utilized the interferon-gamma release assay (IGRA) ELISA method with QuantiFERON-TB Gold Plus (QFT-Plus) kits to quantify the release of interferon-gamma by white blood cells when exposed to Mycobacterium tuberculosis antigens^33^. Following standard procedure, assay results were categorized as indeterminate, negative, or positive. For concise operational guidance on IGRA procedures, a supplementary file containing a brief operations manual has been provided (see Additional file 2). In cases where the initial assay results were indeterminate, a repeat run was conducted in the laboratory using the same ELISA procedures but with a different kit, as obtaining a repeat blood sample from participants was not practically feasible. Individuals testing positive for latent TB infection were excluded from having active TB, while those testing negative for active TB were initiated on TB preventive treatment (TPT). Detailed results and findings of this treatment will be presented in a subsequent publication. In addition to rigorous participant enrollment procedures, our project staff underwent comprehensive training in all aspects of the manual of procedures, including questionnaire administration, data collection techniques, informed consent procedures, and performing the IGRA test using QFT-Plus kits. To ensure participant confidentiality throughout data collection, unique identifiers were assigned. The acquired data was meticulously entered into EpiInfo software version 7 (https://www.cdc.gov/epiinfo/support/downloads.html) and subsequently downloaded as an Excel sheet for rigorous analysis^32^.

#### Qualitative

For the qualitative phase, in-depth interviews were conducted using a structured interview guide (see Additional file 3), with discussions centered on recognizing silica-dust-exposed individuals as a high-risk group for latent tuberculosis infection (LTBI) testing and the challenges associated with LTBI testing in community settings. Experts were approached for participation following a detailed telephonic discussion outlining the study's purpose, benefits, and the investigator's motives. Interviews were scheduled at a time and location convenient for the experts, ensuring their full participation. Notably, each in-depth interview, lasting between 15 and 30 min, was conducted in a one-on-one setting with no third-party presence. The interviews were systematically audio-recorded to capture the nuanced insights shared by the experts. All interviews, recorded in audio, underwent transcription into English in a Word document (see Additional file 4). Although no formal quality checks were in place, the transcriptions were reviewed by the interviewers, who are proficient in both the local language and English. In accordance with the COREQ guidelines, which require verifying the accuracy of transcribed content with the interviewees, the transcripts were shared with the individual interviewees for feedback. Each interviewee was provided with their own transcript, primarily through WhatsApp, and follow-up was conducted via phone calls to confirm any corrections or feedback. This process was intended to ensure that the transcribed text accurately reflected their statements during the interviews. Only one interviewee suggested minor grammatical corrections, which were subsequently incorporated, while the remaining interviewees agreed with the transcript. No additional field notes were generated during the in-depth interviews.

### Study variables (quantitative)

The primary outcome variable was binary, indicating the presence or absence of latent tuberculosis (TB) infection as determined by a positive or negative result on the Interferon Gamma Release Assay (IGRA) test. The predictive variables encompassed a range of factors, including age, gender, years of engagement in agate-stone work, overcrowding (categorized based on recommended occupancy: 2 persons for a single room, escalating to 3, 5, 7, and 10 persons for two to five rooms, respectively)^34^, tobacco smoking (smoked at least 1 bidi/cigarette in the last month), Bacillus Calmette-Guérin (BCG) vaccination status, type of work, history of previous TB treatment, body mass index (BMI), and the standard of living index (SLI). The SLI was computed using asset ownership information (see Additional file 5), with variables selected from the National Family Health Survey 2019–2021 (NFHS-5)^35,36^. The SLI index, derived from these standard references, is categorized as low (1–11), middle (12–22), and high (23–34), providing insights into the participants’ socio-economic status^36^. The variable 'type of work' was categorized into two groups: polishers/chippers and drillers, based on observed differences in the nature of these activities. Polishing and chipping involve larger-sized stones, while drilling entails creating small holes in smaller pieces of agate stones. This categorization aims to capture potential distinctions in occupational activities that may impact the risk of latent TB infection among agate-stone workers, without measuring actual dust concentrations.

### Statistical analysis

#### Quantitative

To conduct the quantitative analysis, the dataset extracted as an Excel sheet from EpiInfo software version 7 (https://www.cdc.gov/epiinfo/support/downloads.html) was imported into the Statistical Package for Social Sciences (SPSS) version 23 (https://www.ibm.com/products/spss-statistics)^32,37^. Categorical variables were succinctly described using percentages, while continuous variables were presented as median with interquartile range (IQR). Initially, univariable logistic regression was employed to identify potential variables for inclusion in the multivariable logistic regression model. Variables with a p-value < 0.2 in the univariable analysis were considered for integration into the multivariable model. Assessment for co-linearity was conducted using tolerance and variance inflation factor (VIF) statistics, where values indicating tolerance < 0.1 and VIF > 10 were considered indicative of co-linearity. Subsequently, multivariable logistic regression was utilized to pinpoint independent and significant predictors of latent TB infection. Adjusted odds ratios along with their 95% confidence intervals (CIs) were computed using this multivariable logistic regression approach. The analysis aimed to uncover relationships between variables and identify independent predictors of latent TB infection. Statistical tests were selected based on the nature of our hypotheses, with significance set at p < 0.05.

#### Qualitative

For the qualitative analysis, the transcript in the Word document was coded using the 'comment' feature in Microsoft Word 2016 (part of Microsoft Office 2016, https://www.microsoft.com/en-us/microsoft-365/previous-versions/microsoft-office-2016). Subsequently, the codes were compiled in a Microsoft Excel 2016 sheet (part of Microsoft Office 2016, https://www.microsoft.com/en-us/microsoft-365/previous-versions/microsoft-office-2016), and similar codes were merged into single categories. Once the codes were finalized, they were organized and grouped into categories through thematic analysis. The primary objective was to explore experts' perceptions regarding LTBI testing in silica-dust-exposed individuals and the associated challenges in community settings. The qualitative analysis process involved a reflexive approach, where the data were coded with a focus on maintaining interpretive depth and rigor. The reflexivity in the analysis enriched the findings. Given the absence of prior qualitative studies on this subject, codes emerged from experts' perceptions and suggestions during the interviews. Using inductive reasoning, the coding process began with open coding, assigning broad codes after breaking down the transcript into smaller segments. These codes were then listed in an Excel sheet, with some being merged or recoded to ensure clarity. Moving forward, axial coding linked open codes to establish relationships, and similar patterns were grouped into categories. The final step, selective coding, identified important themes expressing the essence of the analysis. The resulting categories and themes were instrumental in drawing conclusions. Throughout, any inconsistencies were validated by revisiting the interviews, and no additional data collection was pursued to resolve discrepancies. The analysis, presented in a codebook with a description of each code (see Additional file 6), was subjected to participant feedback and was approved by all study participants of the in-depth interviews without any suggested changes or corrections.

### Ethics approval and consent to participate

This study received approval from the Scientific Advisory Committee (SAC) and the Institutional Human Ethics Committee (IHEC) of the ICMR—National Institute of Occupational Health (Ahmedabad, Gujarat) [Approval Code: ICMR-NIOH/EC/2023/4/3.16, dated February 14, 2023]. Additionally, permission to conduct the study was granted by the State TB Operations Research Committee (Government of Gujarat). Prior to participation, written informed consent was obtained from all individuals involved in the study. This included informed consent from agate-stone workers for their participation in filling out the questionnaire and providing blood samples for latent TB infection testing, as well as from those participating in the in-depth interviews, including their agreement to audio recording. The research adhered to the principles outlined in the Helsinki Declaration. The research team maintained exclusive access to all collected data, and participant identities were safeguarded through the use of unique identifiers.

## Results

### Quantitative

**Figure 1:**
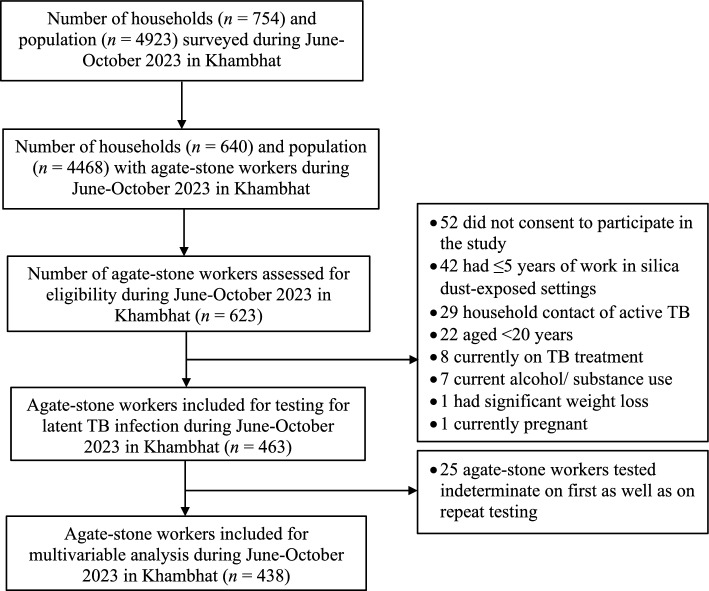
Selection of agate-stone workers for latent TB infection testing during June-October 2023 in Khambhat.

In our survey of 4923 individuals across 754 households in Khambhat, 463 eligible agate-stone workers were identified for latent TB infection testing using IGRA kits (Fig. 1). Remarkably, 52 agate-stone workers (8%) opted not to participate in the study, presenting a challenge in procuring blood samples for the detection of asymptomatic infections.

#### Characteristics of agate-stone workers

The study cohort predominantly comprised males (85%), with a median (IQR) age of 35 (28–45) years, a median (IQR) BMI of 22 (19–26), and a median (IQR) of 6 (3–8) years of formal education (Table 1). Regarding occupational factors, 90% of participants were engaged in cottage industries, and the median (IQR) duration of employment in the agate-stone sector was 12 (8–20) years. Notably, 58% (95% CI 53–63%) of workers tested positive for latent TB infection, as determined by the IGRA results.

**Table 1:** Characteristics of agate-stone workers during June-October 2023 in Khambhat (n = 463).

Characteristics	Number (%) or median (IQR)
Socio-demographic	
Age (years)	35 (28–45)
Male gender	393 (85)
Body mass index (BMI)	22 (19–26)
Single (vs. married)	68 (15)
Formal school education (years)	6 (3–8)
Urban (vs. rural) residence	188 (41)
Extended (vs. nuclear) family	335 (72)
Per-capita monthly income (Indian rupees)	2000 (1333–2500)
Overcrowding	349 (75)
Standard of living (SLI) index	
Low (SLI score of 1–11)	113 (24)
Middle (SLI score of 12–22)	336 (73)
High (SLI score of 23–34)	14 (3)
Tobacco smoking	38 (8)
Tobacco chewing	259 (56)
Work-related	
Work setting	
Cottage	418 (90)
Factory	45 (10)
Work in agate-stone settings (years)	12 (8–20)
Primary work setting vis-à-vis agate stones	
Polishing	301 (65)
Chipping	46 (10)
Drilling	116 (25)
Using water-drip method while working	404 (87)
Using mask while working	1 (0.2)
Clinical	
Vaccinated with BCG	317 (69)
Known diabetic	14 (3)
Previously treated for TB	22 (5)
Immunocompromised state	3 (1)
Interferon gamma release assay (IGRA) result	
Positive (latent TB infection)	269 (58)
Negative	169 (37)
Indeterminate	25 (5)

#### Predictors of latent TB infection

After thorough assessment for co-linearity (see Supplementary Table S5 in Additional file 7) and utilizing a significance threshold of p-value < 0.2 in univariable logistic regression, several variables were considered for further analysis (Table 2), including urban residence, overcrowding, BCG vaccination, a five-year increment in silica-dust-exposed settings, and type of work (polishing/chipping vs. drilling). Among these, only a five-year increment in exposure duration, type of work, and BCG vaccination emerged as significant predictors of latent TB infection. Polishers/chippers exhibited twice the odds (95% CI 1.2–3.2, p = 0.004) of testing positive compared to drillers. Each five-year increase in exposure duration was associated with a 14% rise (95% CI 2–26%, p = 0.016) in the odds of latent TB infection, and BCG vaccination conferred a two-fold increase (95% CI 1.2–2.8, p = 0.008).

**Table 2:** Univariable and multivariable logistic regression for predictors of latent TB infection among agate-stone workers during June–October 2023 in Khambhat (n = 438).

Predictors	Crude OR (95% CI)	p-value	Adjusted OR (95% CI)*	p-value
Age (years)	1.01 (0.99–1.03)	0.251	–	–
Male gender	1.4 (0.8–2.3)	0.253	–	–
Body mass index (BMI)	1.03 (0.98–1.1)	0.232	–	–
Single (vs. married)	1.1 (0.6–1.8)	0.841	–	–
Formal school education (years)	1.0 (0.9–1.1)	0.995	–	–
Urban (vs. rural) residence	1.5 (1.03–2.3)	0.035	1.5 (0.9–2.3)	0.057
Overcrowding	1.5 (0.9–2.3)	0.081	1.4 (0.9–2.2)	0.143
Extended (vs. nuclear) family	0.9 (0.6–1.4)	0.742	–	–
Per-capita monthly income (Indian rupees)	1.0 (0.9–1.01)	0.441	–	–
Standard of living (SLI) index (low vs. middle/ high)	1.3 (0.8–2.1)	0.258	–	–
Tobacco smoking	0.8 (0.4–1.6)	0.534	–	–
Tobacco chewing	0.9 (0.6–1.3)	0.639	–	–
Cottage (vs. factory) work setting	0.7 (0.4–1.5)	0.404	–	–
Work in silica dust-exposed settings (five-year unit increase)	1.1 (0.9–1.2)	0.084	1.14 (1.02–1.26)	**0.016**
Primary work setting vis-à-vis agate stones (polishing-chipping vs. drilling)	1.7 (1.1–2.6)	0.022	2 (1.2–3.2)	**0.004**
Using water-drip method while working	0.9 (0.5–1.7)	0.860	–	–
Vaccinated with BCG	1.5 (0.9–2.2)	0.065	2 (1.2–2.8)	**0.008**
Known diabetic	1.1 (0.3–3.3)	0.933	–	–
Previously treated for tuberculosis	1.9 (0.7–5.3)	0.233	–	–
Immunocompromised state	1.3 (0.1–14.6)	0.825	–	–

*Omnibus test χ^2^ = 23.1, df = 5, p < 0.001; Hosmer–Lemeshow goodness of fit test p = 0.372; Nagelkerke R^2^ = 0.07; classification accuracy = 63.9%.

Significant values are in bold.

### Qualitative

**Figure 2:**
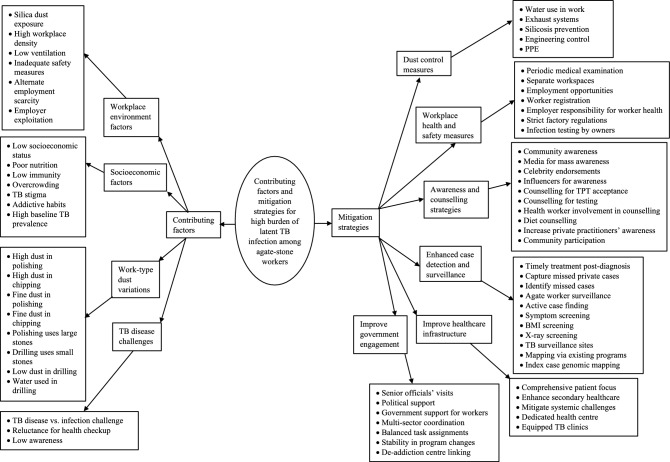
Contributing factors and mitigation strategies for high LTBI burden among agate-stone workers in Khambhat.

**Figure 3:**
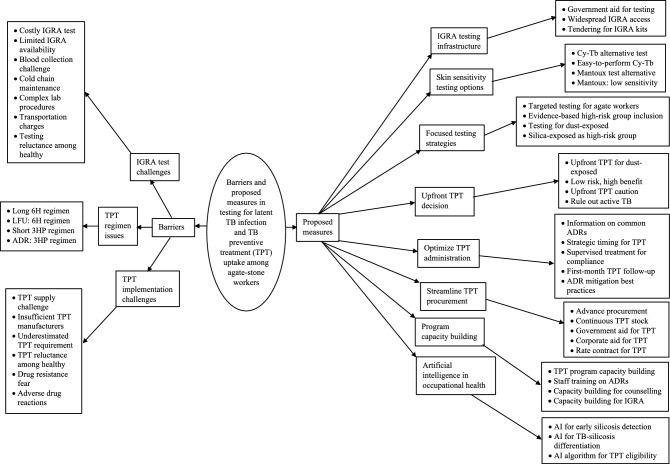
Barriers and proposed measures for improving LTBI testing and TPT implementation among agate-stone workers in Khambhat.

The median (IQR) experience among the thirteen interviewees was 7 (5–10) years, with one female participant. The analysis of codes and categories from the transcripts of in-depth interviews revealed four overarching themes: contributing factors and mitigation strategies for the high burden of latent TB infection (LTBI) (see Fig. 2 and Supplementary Tables S1 and S2 in Additional file 6 for detailed code descriptions); barriers to and proposed measures for LTBI testing and TB preventive treatment uptake among agate-stone workers in Khambhat (see Fig. 3 and Supplementary Tables S3 and S4 in Additional file 6 for detailed code descriptions). Figures 2 and 3 represent code trees derived from this qualitative analysis, illustrating the relationship between the identified codes, categories, and overarching themes. The codebook in Additional file 6 provides detailed descriptions of each code under various categories, further supporting the theoretical framework presented in Figs. 2 and 3.

#### Contributing factors for high LTBI burden

Experts highlighted that the high burden of latent TB infection (LTBI) among agate-stone workers in Khambhat, Gujarat, is attributed to various factors. Prolonged exposure to silica dust in the workplace poses a significant risk, known to contribute to the development of active TB disease. Polishing and chipping activities, characterized by increased dust generation and finer particles, showed higher LTBI positivity compared to drilling. Socioeconomic challenges such as low income, poor nutrition, and overcrowded living conditions further increase susceptibility to LTBI. Additionally, experts pointed out that low awareness regarding the distinction between active TB disease and latent TB infection poses a challenge, potentially impacting testing acceptance and subsequent preventive treatment uptake.

“According to me, there could be 2–3 reasons. Firstly, the patient may have been exposed to dust, which could possibly lead to a decrease in their lung's clearing capacity, thereby potentially causing infection due to exposure to dust or possibly due to their low immunity. Another aspect to consider is the nutrition status of such individuals, especially in workers like these, where we should assess their nutritional status at least once, and linking it with BMI might give us a rough estimate of their actual nutrition status. Another factor could be their socio-economic condition, as it could be a compounding factor. It could be possible that they are working in environments where ventilation is poor or their beliefs and understanding… might not directly contribute to infection but could affect their living conditions, which in turn could affect their infection.” (State-level TB program official, 2 years of experience).

#### Mitigation strategies for high LTBI burden

In response to the high burden of latent tuberculosis infection (LTBI) among agate-stone workers, experts proposed several mitigation strategies. These encompass implementing comprehensive dust control measures within the workplace to mitigate silica dust exposure, bolstering workplace health and safety protocols by introducing infection testing conducted by factory owners, and fostering community-wide awareness campaigns aimed at promoting LTBI testing and TB preventive treatment uptake. Timely initiation of TB preventive treatment following LTBI diagnosis was identified as a critical intervention.

“If we want to reduce the burden of latent TB infection among agate workers, we need to target this particular population. Initially, we should conduct tests for latent TB infection among them, and once the results are available, provide treatment promptly. If we can diagnose them early and provide timely treatment, we can control the spread of infection and prevent further transmission. So, the main focus should be on early diagnosis and timely treatment.” (District lead for TPT program, 7 years of experience).

“Based on my experience conducting any test for any people like TB test or latent TB test or for any test… so these are the special groups where we are just wanted to check their positivity or the prevalence of some of the disease but getting the testing done for normal person who is not having any of the symptoms for anything, it is little tough. So the first point is that counselling is required. Why is this testing to be done and post-testing what could be the conditions where they are required to be on medication or not. So that counselling needs to be done at the first point. The second thing is whenever we ask any patient to be done the test… the fear zone they come across… so because of the fear factor they refuse to do some testing and because of that only the high level of counselling needs to be done for the agate workers. And the local person or some CHC/PHC staff or some government staff can help us to do good amount of testing. ASHA workers also can be very helpful in such case where they convince these kind of workers to do some testing and spreading awareness is the major point where we can convince them for at least testing, for the kind of testing we are going to do.” (State lead for new initiatives, 9 years of experience).

#### Barriers in LTBI testing and TPT implementation

Despite efforts to address LTBI among agate-stone workers, several barriers hinder effective testing and implementation of TB preventive treatment (TPT). Challenges related to IGRA test availability, cost, and complexity pose significant barriers to LTBI testing. Similarly, issues such as limited TPT supply, drug resistance fears, and adverse drug reactions impede the successful implementation of TPT among exposed individuals.

“When it comes to IGRA testing, the cost for conducting one test ranges from 1800 to 2000 [Indian rupees], and even in this, the tests we conduct at the field level require us to work with blood samples. These samples need to be maintained with cold chain maintenance until they reach the designated laboratory, where testing can be done. This process usually takes almost 2 to 3 days, which means 48 to 72 h until we receive the report. All these hurdles are associated with it.” (District-level medical officer, 13 years of experience).

“The challenges in TPT, is the supply of medicines, so it is supplied from the central level, so from the central level, we are given 6H, so the Isoniazid, we have to give for 6 months, and there is an issue in the adherence. We are giving it in contacts of TB patients… generally they say no to take 6H. In private doctors also, there is an issue of acceptance, the private doctor has a fear, that if we give Isoniazid for 6 months, then there will emergence of drug resistance. So we are facing these challenges. And from the national level, the medicines that are supplied, especially in the last one year, there was some constraint. Now in the near future, 3HP will be supplied, which has 12 doses, and these are weekly doses, so chances of acceptance are more. So from the central TB division, if the medicine comes, I think it will be beneficial. So the main challenge is, the contacts of [TB patients in] the private sector, so in that contact, there is less acceptance, because in the child, from 0 to 5 years, when we were giving it to the child, there was no issue of acceptance, as it was a dispersible tablet, it does not affect the child. But in adults when we give TPT in the absence of any symptoms, there is less acceptance. So it is a matter of behaviour change, so the community needs to be aware, along with that, during CMEs, the doctors need to be made aware… even with one-to-one interactions we explain it to the doctors that by giving TPT, there is a reduction in incidence, around 10%, that is what the document says, so it will benefit us.” (State-level TB program official, 10 years of experience).

#### Proposed measures for improving LTBI testing and TPT implementation

To address the challenges in LTBI testing and TPT implementation, experts have proposed several measures. These include enhancing the infrastructure for IGRA testing and ensuring widespread availability of IGRA kits through government support and tendering processes. Moreover, exploring alternative testing options, such as skin sensitivity testing, can provide additional avenues for diagnosing LTBI among agate-stone workers. There is a recommendation to replace 'silicosis' with 'silica-dust exposed' as the high-risk group for testing for LTBI in the national TB program guidelines, reflecting a more accurate representation of the at-risk population. There is a division in opinion regarding the direct administration of TB preventive treatment without LTBI testing, with some experts advocating for such an approach in settings with a high prevalence of LTBI, while others emphasize adherence to established diagnostic algorithms.

“Another thing is that often, the term ‘Silicosis’ has been used less, and the term ‘Dust Exposure’ or wherever dust emanates, if this term is used, then all individuals affected by it, many more populations, which are linked with different occupations, can be included. So, if we test everyone where dust exposure occurs, and check the infection in everyone, and detect infection and give TB preventive treatment, then the incidence of the disease can be reduced…. TB incidence which was previously reduced by around 2% per year, which is around 10–15% currently… I believe we can bring in a reduction of 25% every year in TB incidence. It is very important to pay attention to the high-risk group in our NTEP guidelines… if we pay maximum attention to this high-risk group, then we can reduce the incidence of infection and TB significantly, in my opinion.” (District-level medical officer, 13 years of experience).

“In general population, it is necessary to test everyone, but according to me, for the vulnerable population, for example, contacts of TB patient, or the place where the silica is generated, according to you, more than 60% to 70% of the workers are positive… so when positivity, the prevalence of infection is more in such vulnerable population, in that, instead of test and treat strategy, we can go for direct treat strategy. But, of course, it is necessary to check, whether they have TB or not, and if TB is ruled out, then TPT should be given directly, according to me.” (State-level TB program official, 10 years of experience).

## Discussion

In summary, our findings reveal that among agate-stone workers, the prevalence of latent TB infection exceeded that reported for the general population. Notably, exposure to silica dust emerged as a significant factor, especially prolonged exposure and engagement in specific work settings involving polishing and chipping of agate stones. Experts concurred with these findings and advocated for targeting agate-stone workers and other similarly silica-dust-exposed occupational groups for latent TB testing and subsequent TB preventive treatment, aiming to mitigate the burden of TB within these vulnerable populations.

Several occupational and sociodemographic factors contribute to our study's findings. Exposure to silica dust increases the likelihood of developing active TB disease twofold^38^. Given the high silica content of agate stones, agate-stone workers are particularly susceptible to developing active TB disease^26^. In addition to the risk posed by silica dust exposure, the inherent presence of sociodemographic risk factors for active TB transmission and documented higher baseline TB prevalence among agate-stone workers^28,29^ suggest a potentially elevated burden of latent TB infection in our study population. The observed higher incidence of active TB disease indicates a greater conversion rate from latent TB infection, underscoring the necessity for TB preventive treatment interventions within this community. Following interventions by the National Human Rights Commission in Khambhat^39^, the grinding, a process of shaping beads by abrading against an electrically rotating emery wheel, identified as a leading cause of respiratory morbidity among agate-stone workers^28,29^, was halted. Also, water-drip or water flow was suggested to be incorporated in all possible processes in place currently^22,39^. Thus, in the current scenario, the polishing and drilling processes utilize water—polishing using a water-drip, whereas drilling using a frank water flow—whereas chipping doesn’t utilize water as it is practically unfeasible. Had the grinding process still continued in Khambhat among agate-stone workers, our study would have documented > 70–75% burden of latent TB infection. This explains the comparatively lower prevalence of latent TB infection against expected in high-risk groups such as ours.

The prevalence of latent TB infection among agate-stone workers in our study was 58%, surpassing the 41% reported for high-risk groups^17^, and nearly doubling the nationwide survey's figure (31%) for the general population^18^. Our finding of LTBI prevalence (58%) among agate-stone workers closely aligns with the reported prevalence among coal workers' pneumoconiosis (66%) in China using IGRA^11^, and exceeds the 41% and 47% reported in silicosis and coal miners using IGRA in China and Germany, respectively, in other studies^14,15^. Studies reporting higher LTBI prevalence, such as among South African gold miners (89%)^40^ employed skin sensitivity tests (Mantoux test) rather than IGRA. Conversely, among patients with silicosis, some studies reported lower prevalence using the Mantoux test: 57% in China^15^ and 36% in Sri Lanka^16^. The susceptibility of the Mantoux test to cross-reactions with BCG vaccination, sensitivity to non-tuberculous mycobacteria, the potential for the boosting phenomenon, and subjectivity in interpretation may explain these discrepancies^41,42^. Overall, the prevalence reported in our study was comparable to other studies using the IGRA test for diagnosing LTBI.

In our study, we observed a 14% increase in the likelihood of LTBI positivity with every five-year increment in the number of years worked in the agate-stone industry. While we lack direct comparative evidence for these results, a study among coal miners in Germany identified increased age as a predictive factor, with age serving as a proxy for the duration of dust exposure^14,43^. The finding of BCG vaccination increasing the likelihood of LTBI positivity in our study presents a paradoxical result, as BCG vaccination is known to reduce the risk of both LTBI and active TB among patients with silicosis^15^. One potential explanation could be related to the method of BCG vaccination verification used in our study, which involved confirming the presence of a BCG scar mark during data collection. It's worth noting that not all individuals develop a scar mark despite receiving the vaccine and there may also be issues of observer bias in reading the scar^44,45^. Additionally, this finding raises questions about the effectiveness of BCG vaccination in this specific occupational context, emphasizing the need for further research to elucidate the underlying mechanisms.

Multiple challenges exist regarding the diagnosis, delayed diagnosis, misdiagnosis, and notification/reporting of silicosis in India^22^. Official figures from the Government of India over a fifteen-year span indicate only 441 reported cases of silicosis, according to the Ministry of Labour and Employment^22,46^. However, in 2015–2016 alone, an estimated 11.5 million workers were exposed to silica dust in their occupations in India, a number projected to rise to 52 million by 2025–2026^22,47,48^. Given the recognized risk that exposure to silica dust poses for TB^38^, and in line with the expert opinions documented in our in-depth interviews, we advocate for the inclusion of silica-dust-exposed individuals as a high-risk group in the guidelines for the programmatic management of TB preventive treatment, specifically for testing for latent TB infection. While some guidelines recommend screening workers exposed to silica dust for ≥ 25 years, these are primarily based on standards in the US^49^. In India, where the permissible exposure limit (PEL) for silica dust is much higher than international standards^22^, and silicosis can occur even at the recommended PELs^50^, we argue that silica-dust-exposed workers with ≥ 5 years of exposure should be considered candidates for latent TB infection testing. This recommendation is based on the understanding that silicosis, which also increases susceptibility to TB, typically develops within 5–10 years of exposure^31,51^.

Experts in our study highlighted multiple challenges associated with the interferon-gamma release assay (IGRA), ranging from its costliness to the requirement for blood collection. Consequently, Cy-Tb was proposed as an alternative^52^. Cy-Tb, previously known as C-Tb, is a novel specific skin test based on ESAT-6 and CFP10 antigens^53^, and has been approved by the Indian Council of Medical Research (ICMR) for the diagnosis of latent TB infection in individuals aged ≥ 18 years^54^. Cy-Tb exhibits comparable specificity to IGRA using QFT-Plus (99.3% vs. 98%), although it demonstrates lower sensitivity (74% vs. 91%) and should be considered as a replacement for IGRA^52,53^.

In our study, experts suggested shorter TB preventive treatment (TPT) regimens, such as rifamycin-containing regimens like Isoniazid-Rifapentin, for improved compliance and treatment efficacy. However, the experts also noted that these regimens were associated with higher rates of adverse drug reactions. Similar findings were reported in qualitative research conducted in Canada, as well as in other quantitative studies and a recent meta-analysis^55–57^. In light of the adverse drug reactions associated with Isoniazid-Rifapentin regimen, experts in our study recommended thorough follow-up in the first month, preferably with a directly observed preventive therapy (DOPT) mode of delivery^55,57^. The feasibility of upfront TB preventive treatment (TPT) without testing requires further research in high-burden silica-dust-exposed occupational settings. As noted by experts in our study, in scenarios where three out of four silica-dust-exposed individuals test positive for latent TB infection, testing might be deemed redundant, and a 'direct-treat' strategy could be more appropriate, considering resource constraints, instead of the test-treat approach. However, due to conflicting opinions among interviewees, with some experts advocating for a guideline-driven algorithm-based diagnosis, we recommend generating evidence on this matter before implementing the 'direct-treat' strategy in place of the 'detect-treat-prevent-build' approach recommended under India's national strategic elimination plan^58^. This recommendation aligns with the WHO guidelines, which advocate for at least yearly active TB screening for individuals currently or previously exposed to silica dust, as initiating TPT inherently includes ruling out active TB^59^.

This study represents the first investigation in India to estimate the burden of latent TB infection among silica-dust-exposed workers. The research adheres to the criteria for reporting cross-sectional and qualitative research (see Additional file 8)^60,61^. Agate-stone workers were not included in the interviews as the primary aim of the in-depth interviews was to document programmatic challenges in testing and preventive TB treatment. Given the objective of conducting a thematic analysis and our study being designed to directly address the unique challenges faced by agate-stone workers, the qualitative in-depth interviews were analyzed without a predefined theoretical framework. Regarding participants who tested as indeterminate on IGRA results, obtaining a repeat blood sample was not practically feasible due to the refusal of study participants to provide another blood sample. Consequently, a repeat run was conducted in the laboratory using the same ELISA procedures but with a different kit. The results were then interpreted based on the findings of the second run, ensuring that we had a reliable result for each participant. Nevertheless, even with all these limitations, given the good internal validity and standard procedures followed with good quality checks, we believe that the findings are generalizable to occupational settings similarly exposed to silica dust in India.

## Conclusions

Based on the study’s findings, we conclude that the burden of latent TB infection (LTBI) among agate-stone workers was nearly double that of the general population. Furthermore, occupations involving higher levels of silica dust exposure exhibited a correspondingly higher burden of TB infection, with the likelihood of testing positive for infection increasing in proportion to the duration of exposure. Experts reiterated that these findings hold significant policy implications, particularly regarding the inclusion of silica-dust-exposed individuals as a high-risk group for systematic testing and treatment for latent TB infection in the national TB guidelines of India. Additionally, considering the costliness of current IGRA testing methods, newer techniques such as improved skin sensitivity tests like Cy-Tb should be explored as alternatives. The study also highlighted the potential benefits of shorter TB preventive treatment (TPT) regimens in terms of compliance, albeit with a higher risk of adverse drug reactions compared to longer regimens necessitating supervised treatment and stringent follow-up. For clinical practice, our findings support the implementation of upfront TB preventive treatment for silica-dust-exposed individuals who have worked for ≥ 5 years, without the need for LTBI testing. This approach aims to mitigate the risk of progression to active TB and reduce the overall TB burden in this high-risk population. Future research should aim to establish specific thresholds for silica dust exposure, duration of exposure, and LTBI prevalence that would warrant upfront TB preventive treatment in occupational settings.

### Supplementary Information

Supplementary Information 1.

Supplementary Information 2.

Supplementary Information 3.

Supplementary Information 4.

Supplementary Information 5.

Supplementary Information 6.

Supplementary Information 7.

Supplementary Information 8.

## Data Availability

The quantitative survey data are available from the corresponding author (Dr. Mihir Rupani) upon reasonable request. All data generated or analyzed during the qualitative in-depth interviews are included in this published article and its supplementary information files.
