# Supplementary material for: Mixed methods study on latent tuberculosis among agate stone workers and advocacy for testing silica dust exposed individuals in India
Source: Sci Rep. 2024 Jun 15;14:13830. doi: 10.1038/s41598-024-64837-4 (PMC11180111; doi:10.1038/s41598-024-64837-4)
Supplement: Supplementary file 1 — Supplementary Information 1. [file 41598_2024_64837_MOESM1_ESM.docx]

**Data entry done** Date of entry: __ __ / __ __ / __ __ __ __

d d m m y y y y

**Form 01: Household information**

1. Today’s date: __ __ / __ __ / __ __ __ __

d d m m y y y y

1. Name of village/area: Shakkarpur ☐1 Dadiba ☐ 2 Machipura ☐ 3 Akabarpur ☐ 4

Pirajpur ☐ 5 Other ☐ 6

Any other (specify) ________________________________________

1. Household number: ____ ____ ____ ____
2. Name of head of family: __________________________________________________________________________________
3. Street address (detailed): _______________________________________________________________________________

__________________________________________________________________________________________________________________

1. Total number of family members: ____ ____
2. Any household member working in silica-dust setting (agate-related work)? Yes ☐1 No ☐ 0
   1. If yes, total number of family members working in agate-related work: ____ ____

- ***If yes to Q7, fill Form 02. If no, end the interview appreciating the resident, and visit the next house.***
- ***For >1 person working in agate-related work in a house, fill separate forms for each.***

**Form 02: Participant tracking**

1. Name: First______________________ Middle______________________ Surname_______________________________

2. Mobile phone: Worker ___________________________________ Relative ______________________________________

3. Number of years since working in silica-dust setting (agate-related work): ____ ____ years

4. Is age of the worker ≥20 years? Yes ☐1 No ☐ 0

***If yes to Q4, fill form 03.***

**Form 03: Eligibility questionnaire**

Yes No

1. Working in silica-dust (agate) setting for ≤8 years? ……...☐1 ☐ 0 **(if yes, cancel this worker)**
2. Household (close) contact of active TB? ……………………….☐1 ☐ 0 **(if yes, cancel this worker)**
3. Is the worker’s age <20 years?......................................................☐1 ☐ 0 **(if yes, cancel this worker)**
4. Do you suffer from cough for ≥2 weeks? ..................................☐1 ☐ 0 **(if yes, cancel this worker)**
5. Do you suffer from fever for ≥2 weeks? ....................................☐1 ☐ 0 **(if yes, cancel this worker)**
6. Do you suffer from significant weight loss?.............................☐1 ☐ 0 **(if yes, cancel this worker)**
7. Do you suffer from hemoptysis (blood in sputum)?............☐1 ☐ 0 **(if yes, cancel this worker)**
8. Currently on treatment for TB? …………………………………..☐1 ☐ 0 **(if yes, cancel this worker)**
9. Known allergy or hypersensitivity to TB drugs? .…………...☐1 ☐ 0 **(if yes, cancel this worker)**
10. HIV +ve (ask accompanying health worker to help) ...........☐1 ☐ 0 **(if yes, cancel this worker)**
11. Currently on treatment for HIV (anti-retroviral drugs).....☐1 ☐ 0 **(if yes, cancel this worker)**
12. If female and married, currently pregnant? ….…...................☐1 ☐ 0 **(if yes, cancel this worker)**
13. Current use of alcohol?..................................……………….............☐1 ☐ 0 **(if yes, cancel this worker)**

***Participant is eligible only if questions #1 to #13 are no. Explain and administer informed consent.***

1. Did worker agree to participate? ………………………………… ☐1 ☐ 0 **(if no, cancel this worker)**

***If yes to Q14,* CAPTURE GEOLOCATION IN KOBOCOLLECT**

***After capturing geo-location, fill the following forms.***

**Data entry done** Date of entry: __ __ / __ __ / __ __ __ __

d d m m y y y y

**Form 04: Socio-demographic and clinical history**

1. Household number (same as Form 01): ____ ____ ____ ____
2. Participant study ID: NIOH-LTBI - ____ ____ ____ ____
3. Date of interview: __ __ / __ __ / __ __ __ __

d d m m y y y y

1. What is the age of the study participant? ___ ___ years
2. Gender: Male ⎕ 1 Female ⎕ 0
3. Marital status: Married ⎕ 1 Unmarried ⎕ 2 Divorced ⎕ 3 Widow/widower ⎕ 4
4. Educational status: Formal education (attended school) ………………….⎕ 1

No formal education (did not attend school)…………………………...….⎕ 0

1. Total number of years of education (calculate from 1^st^ standard till now): ___ ___ years
2. Are parents/grandparents staying with you? (type of family): Extended ⎕ 1 Nuclear ⎕ 0
3. Do you stay in an urban area or a rural area? Urban ⎕ 1 Rural ⎕ 0
4. Assess the dwelling of the study participant (do not ask): Slum ⎕ 1 Non-slum ⎕ 0
5. Have you smoked bidi/cigarette for >0 days in a week in the last month? Yes ⎕ 1 No ⎕ 0
6. Have you chewed tobacco product for >0 days in a week in the last month? Yes ⎕ 1 No ⎕ 0
7. Current use of illicit drugs/substance use? Yes ☐1 No ☐ 0
8. Are you suffering from diabetes? Yes ⎕ 1 No ⎕ 0 Don’t know ⎕ 3
9. Are you vaccinated with BCG vaccine (look for scar on left arm)? Yes ⎕ 1 No ⎕ 0
10. Were you previously affected with tuberculosis? Yes ⎕ 1 No ⎕ 0
    1. If yes, were you previously treated for tuberculosis? Yes ⎕ 1 No ⎕ 0
11. Have you ever been diagnosed with silicosis? Yes ⎕ 1 No ⎕ 0
12. Are you suffering from any of these chronic diseases? COPD ⎕ 1 Asthma ⎕ 2 Cancer ⎕ 3

Heart disease ⎕ 4 Rheumatoid arthritis ⎕ 5 No chronic disease ⎕ 6

1. Does any of these immunocompromised state describe your current condition?

Recent major surgery ⎕ 1 Recent hospitalization ⎕ 2 Prolonged antibiotic treatment ⎕ 3

None of the above ⎕ 4

1. What is your average monthly income? INR ________________________________
2. What is your family’s average monthly income? INR ________________________________
3. Describe your past and current work:

| Workplace | Setting:  cottage - 1/ industry - 2 | Primary work:  Polishing/ drilling - 1,  Crushing/ grinding - 2,  Baking - 3, any other - 4 (specify) | In what activity were you exposed to dust | Number of years in the work setting |
| --- | --- | --- | --- | --- |
| Current |  |  |  |  |
| Past - 1 |  |  |  |  |
| Past - 2 |  |  |  |  |
| Past - 3 |  |  |  |  |
| Past - 4 |  |  |  |  |
| Past - 5 |  |  |  |  |

1. Do you work on agate stones using water-drip method? Yes ⎕ 1 No ⎕ 0
2. Do you wear a mask as a personal protective equipment while working? Yes ⎕ 1 No ⎕ 0

**Examination of study participant:**

1. Height of the study participant: ___ ___ ___ centimeters
2. Weight of the study participant: ___ ___ ___ kilograms

**Form 05: Questions on housing**

1. What type of house do you stay in? Kaccha ⎕ 1 Pucca ⎕ 2 Semi-pucca ⎕ 3
2. How many number of rooms in your house? ___ ___ rooms
3. Does any member of this household own this house or any other house? Yes ⎕ 1 No ⎕ 0
4. Do you own land usable for agriculture? Yes ⎕ 1 No ⎕ 0
5. Do you own livestock, herd or farm animals? Yes ⎕ 1 No ⎕ 0
6. What is your primary source of drinking water? Piped water ⎕ 1

Bore-well water ⎕ 2

Dug-well water ⎕ 3

Other ⎕ 4

Specify _______________________________

1. What is the type of toilet facility at your home? Flush toilet ⎕ 1

Pit toilet latrine ⎕ 2

No facility ⎕ 3

Other ⎕ 4

Specify _______________________________

1. Do you have a separate kitchen in your house? Yes ⎕ 1 No ⎕ 0
2. What is the type of cooking fuel used in your house?

LPG/natural gas……………………………………………………………………………… ⎕ 1

Burning biomass fuel (wood, charcoal, dung and crop residues)……….. ⎕ 2

Other ⎕ 3

Specify _______________________________

1. Does your household have the following facilities:

| **Name of facility** | **Yes / No** |
| --- | --- |
| Television | Yes ⎕ 1 No ⎕ 0 |
| Air conditioner/air cooler | Yes ⎕ 1 No ⎕ 0 |
| Refrigerator | Yes ⎕ 1 No ⎕ 0 |
| Washing machine | Yes ⎕ 1 No ⎕ 0 |
| Bicycle | Yes ⎕ 1 No ⎕ 0 |
| Motorcycle / scooter | Yes ⎕ 1 No ⎕ 0 |
| Car / truck / tractor | Yes ⎕ 1 No ⎕ 0 |
| Mobile | Yes ⎕ 1 No ⎕ 0 |

**Form 06: Latent TB infection**

1. Date of sample collection for IGRA: __ __ / __ __ / __ __ __ __

d d m m y y y y

1. Date of reporting of IGRA test: __ __ / __ __ / __ __ __ __

d d m m y y y y

1. Result of IGRA test: Positive ⎕ 1

Negative ⎕ 0

Name of person who filled the form: _________________________________________________
