# Supplementary material for: Mixed methods study on latent tuberculosis among agate stone workers and advocacy for testing silica dust exposed individuals in India
Source: Sci Rep. 2024 Jun 15;14:13830. doi: 10.1038/s41598-024-64837-4 (PMC11180111; doi:10.1038/s41598-024-64837-4)
Supplement: Supplementary file 2 — Supplementary Information 2. [file 41598_2024_64837_MOESM2_ESM.docx]

**Short standard operating procedure (SOP) for IGRA testing using QFT-Plus kits**

**PHASE 1:**

1. **Blood collection**

Only use lithium heparin as a blood anticoagulant. Label the tube with the Name, HH ID and Participant ID to avoid any mistake. Blood collection tubes should be at room temperature 17–25°C at the time of blood collection. Fill a lithium-heparin blood collection tube (min. volume 5 ml) and gently mix by inverting the tube several times to dissolve the heparin. The tubes must be kept at room temperature between 15 minutes and 3 hours after collection then you can store it in Ice Box for <12 hours or you can directly transfer it to nearby Community Health Centre as the blood samples must be stored at 2-8°C for 16 to 48 hours. After completion of all sample collection put all collected samples into Ice Box and transport it from Khambhat to Ahmedabad NIOH for further processing.

1. **Blood transfer**

The lithium-heparin tube must equilibrate to room temperature at 17–25°C prior to transfer to QFT-Plus Blood Collection Tubes and also allow QFT tubes to equilibrate at room temperature prior to transfer at least for 1 hour. QFT-Plus Tubes must be labelled by participant’s ID (Nil, Tb1, Tb2, Mitogen). Samples must be evenly mixed by gentle inversion before dispensing into QFT-Plus Tubes. Dispense 1 ml of the whole blood specimen into each of the QFT-Plus tubes (Nil, Tb1, Tb2, Mitogen). Mix tubes, immediately after filling the QFT-Plus Tubes, shake them ten times just firmly enough to make sure the entire inner surface of the tube is coated with blood. This will dissolve antigens on tube walls. Then Aliquoted QFT-Plus tubes must be placed in the 37 ℃ incubator within 2 hours of blood transfer and incubate the tubes in an upright position for 16-24 hours.

Note: The total time from blood drawn into heparin tube to 37°C incubation must not exceed 53 hours.

1. **Plasma harvest**

After incubation, centrifuge the specimens at 2000 to 3000 rpm for 15 minutes to separate plasma and red blood cells. The gel plug will separate the cells from the plasma. If this does not occur, the tubes should be re-centrifuged. Harvest the plasma using a pipette.

**PHASE 2:**

1. **Reagent preparation**

Allow all reagents (except conjugate 100x) and specimens to reach room temperature (18°C to 30°C) for at least 60 minutes. After that add 2.280 ml distilled or deionized water to the standard ampule. Now takeout the conjugate from refrigerator and 300 µl distilled or deionized water to the conjugate 100x Concentrate vial, according to the volume printed on the label of the both vial to reconstitute it. Gently mix until it is homogeneously dissolved.

Prepare a series of two-fold dilutions using the Standard Diluent. Label 4 tubes: S1, S2, S3, S4. Add 150 μl of Green Diluent to S1, S2, S3, S4. Add 150 μl of the kit standard to S1 and mix thoroughly. Transfer 50 μl from S1 to S2 and mix thoroughly. Transfer 50 μl from S2 to S3 and mix thoroughly. GD alone serves as the zero standard (S4). The final volume and concentrations of the standard samples should be S1 contains 250 μl (4.0 IU/ml), S2 contains 150 μl (1.0 IU/ml), S3 contains 200 μl (0.25 IU/ml) and S4 contains 150 μl (0 IU/ml) GD alone.

Working strength conjugate is prepared by diluting the required amount of reconstituted Conjugate 100x Concentrate in Green Diluent based on number of strips required. For 12 strips (1 plate ELISA) dilute 60 μl volume of conjugate 100x concentrate in 6.0 ml of volume of Green Diluent.

1. **Perform the IGRA ELISA**

Take 96-well ELISA plate and 1^st^ step is to pour 50 μl working conjugate to all wells. Now add 50 μl of test plasma sample to appropriate wells. Finally, add 50 μl each of the Standards 1 to 4 to the appropriate plate wells. The standards should be assayed in at least duplicate. Cover ELISA plate and mix the conjugate and plasma samples/standards thoroughly for 1 minute. Avoid splashing and incubate it at room temperature for 2 hours. ELISA plate should not be exposed to direct sunlight during incubation. During the ELISA plate incubation prepare working strength wash buffer. For 1 litre dilute 50 ml Wash Buffer 20x Concentrate with 950 ml deionized or distilled water and mix thoroughly. When ELISA plate incubation is complete, wash ELISA plate wells with 400 μl of working strength wash buffer. Perform wash step at least 6 times. A soak period of at least 5 seconds between each cycle is recommended. Kindly wash the plate thoroughly its mandatory. Tap ELISA plate face down on absorbent towel to remove residual wash buffer. Add 100 μl of Enzyme Substrate Solution to each plate well, cover the plate and mix thoroughly for 1 minute and incubate at room temperature for 30 minutes. Do not Exposed ELISA plate to direct sunlight. Following the 30-minute incubation, add 50 μl of Enzyme Stopping Solution to each plate well in the same order as the substrate was added and mix. Measure the Optical Density (OD) of ELISA plate wells within 5 minutes of stopping the reaction using a microplate reader fitted with a 450 nm filter and with a 620 nm to 650 nm reference filter. OD values are used to calculate results.
