# Supplementary material for: Mixed methods study on latent tuberculosis among agate stone workers and advocacy for testing silica dust exposed individuals in India
Source: Sci Rep. 2024 Jun 15;14:13830. doi: 10.1038/s41598-024-64837-4 (PMC11180111; doi:10.1038/s41598-024-64837-4)
Supplement: Supplementary file 3 — Supplementary Information 3. [file 41598_2024_64837_MOESM3_ESM.docx]

**IN-DEPTH INTERVIEW GUIDE**

**Title: Exploring Perceptions and Challenges in Tuberculosis Prevention among Agate-stone workers: A Program Functionaries' Perspective**

**Introduction:** *Greetings and Introduction*

- **Introduction to the Study:**
  - Introduce the study titled "Latent tuberculosis infection and outcomes of tuberculosis preventive treatment among workers exposed to silica dust under programmatic settings."
  - Explain the purpose of the interview: to understand program functionaries' perceptions of the high positivity of latent TB infection among agate-stone workers and the challenges faced in addressing this issue.
- **Informed Consent:**
  - Briefly explain the purpose of the interview and seek consent for participation.

**Section 1: Understanding Perceptions and Reasons for High LTBI Positivity**

1.1 **Perceptions of Program Functionaries:**

- What are your perceptions regarding the significantly higher prevalence (**58%**) of latent TB infection among agate-stone workers compared to the general population (31%)? What explains the near doubling of LTBI in this group?

1.2 **Reasons for High Positivity:**

- In your opinion, what factors contribute to the high positivity of latent TB infection among agate-stone workers, especially those engaged in high silica dust work like polishing or grinding (**2 times higher**) than micro-perforation (**64% vs. 51%**)?
- Do you think there are specific workplace conditions or practices that might explain this phenomenon?

**Section 2: Challenges Faced by Program Functionaries**

2.1 **Challenges in LTBI Testing:**

- Could you elaborate on the challenges faced in conducting LTBI testing among agate-stone workers, considering factors such as the high cost of kits and the need for sophisticated laboratories?

2.2 **Challenges in Tuberculosis Preventive Treatment (TPT):**

- What challenges do program functionaries face in providing Tuberculosis Preventive Treatment to agate-stone workers, especially in terms of the availability of Isoniazid and combination drugs like Isoniazid-Rifapentin?

**Section 3: Solutions and Recommendations**

3.1 **Solutions to Reduce LTBI Burden:**

- According to your experience, what potential solutions can be implemented to reduce the burden of LTBI among agate-stone workers?
- Are there specific interventions or policies that, in your opinion, could effectively address this issue?

3.2 **Role of Tuberculosis Preventive Treatment (TPT):**

- Considering the challenges in TPT availability, how do you think TPT can be effectively utilized for agate-stone workers?
- Are there lessons from the TPT program for household contacts (currently ongoing program being managed by Alert India NGO) that can be applied to agate-stone workers?

**Section 4: Recommendations for National TB Elimination Program**

4.1 **Steps for National TB Elimination Program:**

- What steps do you believe the National TB Elimination Program should take to specifically address the latent TB concerns among agate-stone workers in Khambhat?
- Are there specific policy changes or resource allocations that could facilitate these steps?

**Section 5: Additional probes**

**How can we do awareness generation in a better way so that mass awareness can come?**

**What are the potential solutions to reduce the burden of LTBI among agate-stone workers?**

**What is your opinion on silica-dust-exposed individuals as a high-risk group under the NTEP-PMTPT program, instead of silicosis?**

**In household contacts TPT program, we rule out active TB by X-ray and symptoms. Can the same strategy be used for silica-dust-exposed population?**

**Section 6: Exit question**

**Is there anything else you would like to say that you feel you were not able to say during the interview?**

**Conclusion:**

- Thank the participant for their insights and time.
- Reiterate the importance of their contribution to the study.
