# Supplementary material for: Mixed methods study on latent tuberculosis among agate stone workers and advocacy for testing silica dust exposed individuals in India
Source: Sci Rep. 2024 Jun 15;14:13830. doi: 10.1038/s41598-024-64837-4 (PMC11180111; doi:10.1038/s41598-024-64837-4)
Supplement: Supplementary file 4 — Supplementary Information 4. [file 41598_2024_64837_MOESM4_ESM.docx]

**TRANSCRIPT**

**In-depth interview 1: TB health visitor (TB health worker Khambhat), 5 years of experience**

**What are your perceptions regarding the significantly higher prevalence (58%) of latent TB infection among agate workers compared to the general population (31%)? What explains the near doubling of LTBI in this group?**

The main reason behind increasing in the percentage of latent tuberculosis infection may be, those who are agate workers do not take precautions, they do not wear masks and hand gloves while working. Those who work in the factory, the owners of that factory do not provide them such precaution like masks, glasses for eye protection or any other necessary items, which results in increase in the infection. There is not enough ventilation in a private factory at worker’s house (cottage), due to which dust is flying around in the factory which affects their breathing. In some cases, it can also increase because of addiction of smoking, alcohol or tobacco consumption or use of any illicit drugs. They are not taking nutritional diet, as they themselves came from poverty, housing is overcrowded due to which there is no cleanliness. There is no dust collection system in factories so dust flies and deposits on the road. If they are polishing with the water method, dust is dumped on the roads, which dries up in 2-3 days and dust flies again due to which it again goes into their body by breathing, meanwhile the infection increases in the body. Another thing is that the awareness is very low among these workers. The awareness program is done by the health department the aim of the program is to create awareness among the entire population but all of that is limited as long as those people are present there, as soon as they left the awareness program they forget everything. They do not take any initiative to talk about any kind of problem related to silicosis, tuberculosis or any other respiratory problem. No one is willing to do medical check-up for their health and even think to do that, they are taking it very lightly. As they came from poor family money is more important than health for them. Due to financial issues, they are more likely to do work in such occupations.

**In your opinion, what factors contribute to the high positivity of latent TB infection among agate workers, especially those engaged in high silica dust work like polishing or chipping (2 times higher) than drilling (65% vs. 52%)?**

In the work of Polishing dust likely to fly more, in this work water drip method is uses. But it is not so effective. In the work of drilling, very small hole has to be made in which drip method is also used. In this process less dust flies as compare to polishing and chipping So, there is less chances of getting infection of Latent TB in the process of drilling and higher chances of getting infected in Polishing and chipping.

**Could you elaborate on the challenges faced in conducting LTBI testing among agate workers, considering factors such as the high cost of kits and the need for sophisticated laboratories?**

If we want to Include this in NTEP Programme, we have to Increase the awareness regarding tuberculosis, provide them Mask, Goggles, etc. for Precaution.

**What challenges do program functionaries face in providing Tuberculosis Preventive Treatment to agate workers, especially in terms of the availability of Isoniazid and combination drugs like Isoniazid-Rifapentin?**

Yes, sir there may be some challenge due to insufficient stock of medicines in TPT. Now it is a district level question but after talking to the DTO sir in the state and according to the program, as many patients we can order their medicines in advance or if there is an arrangement to purchase it from any pharmaceutical company, it would be better for us.

**Are there lessons from the TPT program for household contacts (currently ongoing program being managed by Alert India NGO) that can be applied to agate workers?**

The most challenging part of this TPT program is we have to convince their household to take TPT which is most difficult thing to handle. The side effects they get from TPT drugs due to which they are not willing to cooperate. Those people are like we don't have TB then why we have to take the TB medicine. There is miscommunication in explaining all that. But if the arrange Gram Sabha program or any health check-up fairs in which the patient will be handle by medical officer or knowledgeable person kind of public speaker, then it will be better for people to understand that this is not TB but an infection of TB. It would be better if they will understand that we will provide them is not a medicine of TB but a precautionary medicine to prevent TB infection.

**What steps do you believe the National TB Elimination Program should take to specifically address the latent TB concerns among agate workers in Khambhat? Are there specific policy changes or resource allocations that could facilitate these steps?**

A message can be passed that all the factory owners should provide good ventilation facilities in the factory, all the guidelines should be provided to them to provide necessary things like mask, goggles as a precaution. They should be insisted to do Compulsory Health Check-up every 6 months, all of which will be provided free of cost in our government hospitals. Wherever these people work, there is a society, they have their own house or someone's factory, they should be provided with a land at some distance from where they can continue their work and work on the product they are making so that residential guidelines can be given to people so that they do not suffer. We can also provide them a subsidy like things.

**Is there anything else you would like to say that you feel you were not able to say during the interview?**

No, all my points are covered here.

**In-depth interview 2: Medical officer (Khambhat taluka), 7 years of experience**

**What are your perceptions regarding the significantly higher prevalence (58%) of latent TB infection among agate workers compared to the general population (31%)? What explains the near doubling of LTBI in this group?**

First of all, the class we have chosen among the workers of agate, the agate workers are already exposed to silica dust, so there are almost double chances of getting LTBI in those people. Secondly, there is no awareness at all. Their employers, under whom these people work, are not provided with any personal equipment or protective things and they do not even have the knowledge that doing this work can cause this problem. Or harm them in any way. We have seen many challenges at the field level. Those people were not ready to give blood sample, even for X-ray we went to their house and digital X-ray was provided. Then only we could do sample collection, sputum and x-ray. So community awareness is one of the most important issues for me. The community needs to be aware of everything and my suggestions to provide them protective equipment so that any reduction can be seen in this 58% prevalence.

**You have been working in Khambhat for so many years, if you are from Khambhat, what reasons do you think there is such a high prevalence of LTBI? Is it their work related or their occupation related or their sociodemographic that makes them more susceptible to LTBI positivity?**

First thing is that in all the agate workers you have surveyed, there are 2 main communities, the first one is Chunara and the second one is Muslims who work as Agate workers, in addition their residential area is overcrowding, dense. So even in a small house 5 people, even 10 people are working, so such a spacious place is not available. Secondly, those people have been working as labourers for so many years, so now their financial condition is a bit better, but there are many families who are not financially strong. One of my attention is that the issue is in the community itself. There is no community awareness and those people are not financially strong enough to bring equipment for themselves or to do the work anywhere in a good open space. And the area in which all the Agate workers are stay is a high density work place where further exposure to silica is very high which also affects their neighbours around there.

**In your opinion, what factors contribute to the high positivity of latent TB infection among agate workers, especially those engaged in high silica dust work like polishing or chipping (2 times higher) than drilling (65% vs. 52%)?**

The work of drilling in Nagara is done with the help of water flow. It is a simple method, there is no dust exposure and this drilling work is not done anywhere else except Nagara.

**Could you elaborate on the challenges faced in conducting LTBI testing among agate workers, considering factors such as the high cost of kits and the need for sophisticated laboratories?**

The facility of IGRA testing for latent TB should be available, every district should be involved to do this testing, cost of this is around 2000-2500, so the government should have this facility for the community. I only want to say that there should be more awareness programmes organized in the community. It is good for them, for us and for everyone that people know all things and give their consent to do IGRA testing, but due to lack of awareness, people are not ready to do this.

**What challenges do program functionaries face in providing Tuberculosis Preventive Treatment to agate workers, especially in terms of the availability of Isoniazid and combination drugs like Isoniazid-Rifapentin?**

Main issue is the stock of TPT, it is necessary whether the stock of TPT is available in sufficient quantity or not because if it is not in sufficient quantity then it can create an issue and then the second point is to manage all those close contact of the TB patient. We have to give TPT to them but those who have close contact mostly denied to take TPT. Our TB staff works very hard behind them, explains them a lot, but there is one thing in their mind that TB has not happened to me and I am not infected, I am healthy person then why should I take this medicine. There is the main need of awareness. If we have TPT stock available, then we should start this TPT to every worker with silica dust exposure, there is no need for IGRA testing for them and our work also becomes much easier.

**Now let us move towards some solutions, further we have seen challenges which are TPT unavailability, cost of IGRA testing, according to what you said, if we start giving direct TPT instead of IGRA testing, then it will be beneficial for us, apart from that, what are those other potential solutions by which we can reduce the burden of LTBI?**

There is a solution to this that as many agate workers as there are working in the factory, the registration of every worker should be made compulsory, they should also be registered in the municipality. Rules and regulations should be made stricter; factory owners employ poor workers but do not contribute to their health. Protective equipment should be provided in the factory, IGRA testing should be made compulsory for every worker. Moreover, whether TPT should be given or not, the owner of the factory should take care of everything. If all this is done in the factory as a baseline, then there will not be so much load on the government and there is no need for the government to take unnecessary burden. The inspectors of the factory need to bring some strict regulations, because the power they have is not even possessed by the municipality or the health department. Therefore, if everything comes across the health department, then they need more importance and all other departments also need to actively participate in this matter.

**Are there lessons from the TPT program for household contacts (currently ongoing program being managed by Alert India NGO) that can be applied to agate workers?**

For TPT program, TPT should be given to all the silica dust exposed workers. IGRA testing is not necessary because findings already say that people who are continuously in contact with silica dust it affects their lungs directly and weaken the respiratory pathway, so there is no need to IGRA testing as its costly we can directly jump on to give them TPT and then after 6 months or 12 months we will evolve what is in it and how is it, that would be helpful in further studies. If the strategy is completed well, then we can successfully overcome any other challenges.

**What steps do you believe the National TB Elimination Program should take to specifically address the latent TB concerns among agate workers in Khambhat? Are there specific policy changes or resource allocations that could facilitate these steps?**

All I want to say is that political will is very necessary for TB elimination. The message of PM Modi is that to eliminate TB from India by 2025 but political support is very much needed in this matter, and it is more needed for agate workers. If there is support from the political leader according to the area in Khambhat, it can be helpful in spreading awareness in the community. Meanwhile unavailability of TPT also affects it negatively, for that regularly stock should be maintained, if the stock fulfilment is correct then at any time we can convince the workers for TPT by counselling them. Furthermore, factory owners need to bring compulsion in few rules and regulations. Inspectors and coordinators will work together only if we can see significant results. If the health department takes all the burden of TB elimination on itself, no results will come. Every department should work together according to the policy.

**How can we do awareness generation in a better way so that mass awareness can come?**

In the agate workers we are talking about, there are 2 main communities in which you did the survey, are Chunara and Muslims. Within Khambhat, the public of both these communities are seen more and it is more necessary to spread awareness in both these communities. There is overall awareness, it is not like there is no awareness in all communities, but the agate workers are mostly from these 2 communities and the awareness is less in this community. Secondly, in any other financial schemes of the government, I have observed one thing that in finance related schemes everyone participates and everyone cooperates to get benefits. But if there is any health-related program in which good policy is implemented, no one participates. There is a greater need for everyone's co-operation but in that case no one is showing interest which is not good.

**Is there anything else you would like to say that you feel you were not able to say during the interview?**

No

**In-depth Interview 3: Senior treatment supervisor (TB health worker Khambhat), 14 years of experience**

**What are your perceptions regarding the significantly higher prevalence (58%) of latent TB infection among agate workers compared to the general population (31%)? What explains the near doubling of LTBI in this group?**

We have observed a 58% latent TB positivity rate among agate workers. The primary reason for this could be the excessive exposure to silica dust during polishing work in agate factories. Silica dust has a significant impact on these workers' health. If we can prevent the exposure to dust by implementing effective measures, we can significantly reduce the spread of this disease and contribute to its prevention. These workers engage in agate work mainly because they are economically disadvantaged. Poverty is primarily due to the lack of employment opportunities. About 80-85% of silicosis patients come from impoverished households. The owners of these factories, who employ silicosis patients, are the ones making significant profits. Unfortunately, these workers are not paid much attention to. They work for wages throughout their lives and can't progress further. They remain labourers. They are often exploited by factory owners. They have to work in such factories because they cannot find any other employment opportunities.

Regarding the spread of silicosis, it's natural for those who work to get affected, but even those who live with them can contract silicosis due to the dust particles entering their lungs. Silicosis develops slowly over time, and its symptoms can be seen in X-rays. the main reason behind all this is the lack of employment in their homes. If we can provide them with employment, we can prevent them from being affected. If we maintain the same agate business, we need to take precautions. When going to work in factories, workers should undergo mandatory health checks, including medical check-ups, X-rays, TRUNAAT tests, sputum samples, and IGRA tests, to confirm that none of them are infected. If any infection is detected, both the factory owners and the government should take responsibility. The government should provide them with medicine and treatment. If a patient die, their families should also be supported. Thus, by taking some preventive measures, we can ensure the safety of those working at home. For this, fans should be installed in homes, exhaust fans should be used, and if installed, dust should be expelled outside. Expelling dust outside means the infection remains inside. However, if it settles outside, it's safer inside.

Therefore, these people should work with water, which is much safer. By doing so, we can minimize the spread of infection. We need to provide separate spaces for these people to work in their homes. If the government provides support, we can ensure that they have proper facilities. We need to create a system that keeps us informed so that we know when TB patients are being treated. A TB clinic should be set up within a 5 to 10 kilometre radius of every village, where only those who work inside should be allowed entry. Others should not be allowed, so that the spread can be minimized. Moreover, chest X-rays should be done every month, and a TB clinic should be open to provide treatment. If the government supports us a little, the TB clinic will be up and running again. Here, we get facilities for X-rays. Thus, we should guide silicosis patients, provide them with medicines, and work directly under supervision. There should be only one TB clinic centre in each area where patients are screened. Those who test positive are referred to other PHCs, and then to other districts. There, people don't say they have taken medicine. Instead, they generate a new number. This means we have to spend money even though we face difficulties.

**In your opinion, what factors contribute to the high positivity of latent TB infection among agate workers, especially those engaged in high silica dust work like polishing or chipping (2 times higher) than drilling (65% vs. 52%)?**

In chipping work, when the stones are small, the dust-spreading area is also small. This is even smaller in drilling. However, when polishers use larger stones, like volleyballs, the dust-spreading area becomes larger. The chipping process is visible and involves a dripping method, leading to dust falling below. This is different from the past when the surface was smaller, resulting in less visibility during chipping. I don't see any other significant reasons.

**Could you elaborate on the challenges faced in conducting LTBI testing among agate workers, considering factors such as the high cost of kits and the need for sophisticated laboratories?**

If a person needs to undergo an IGRA test, truly these people have to collect blood for the IGRA test, but these individuals do not feel comfortable having their blood collected. Because they are afraid, and when these people work in such factories, the factory owners have IGRA testing certificates for these people, and if it is negative, then only these people are allowed to work. If this is the case, then only these people can have IGRA testing done. That is why we need to bring such a kit to the TB clinic so that the lab technician can conduct IGRA testing. And then we should start TPT for those who test positive. But sometimes TPT is not available. The government's first priority is to provide medicines and give medicine to the patient; this is a major issue. The rest is very difficult to explain. These patients are explained even then, even though our staff explains to them. And among them, 80% believe, and 20% are not ready to understand. Sometimes, senior officials get involved, and they explain to these people because senior officials sit in the office and collect data, but if they have to go to the field, they have to go to the field, like how to explain to a health worker in the field. Understand what STS is, what TBHV is, how counselling is done. If STS and TBHV explain now and the patient does not understand, then it becomes the responsibility of the higher officials to explain to them, and if they still do not understand, then someone else can explain. So, with full police protection and forcefully start treatment, this problem can be resolved. Otherwise, I do not see any other option.

**Are there lessons from the TPT program for household contacts (currently ongoing program being managed by Alert India NGO) that can be applied to agate workers?**

When we implement the TPT program, we may need to explain to people in these households that although they are not ready for X-rays, 80% of them understand, and only 5 to 7 people out of 10 are not prepared. These people may be willing to undergo TB testing if TB medications become available. It is the responsibility of the factory owners to provide TPT to those who have silicosis, and those who go to the factories should be responsible for providing TPT. If we do this under supervision, it will be very easy. Otherwise, we should provide it, and those who understand will take it, but those who do not understand will take it under supervision.

**How can we do awareness generation in a better way so that mass awareness can come?**

So, if we promote awareness in the same way we do for mosquitoes for malaria, even at the village level, and advertise nationally through TV, radio, and other mediums like Amitabh Bachchan does for cough and TB advertisements, then everyone can be involved in this, and a campaign can be run on TV and radio. If someone, through a clip on YouTube, suggests an advertisement for the benefit of all states regarding TB, then the government can collaborate with the TB health department's sponsored programs, providing significant assistance to employees associated with it. Even if an election campaign promotes it and if GSRTC buses display logos and pictures of the Prime Minister, it would greatly help in advertising for TB-free India on TV.

**There are social forces that contribute to the increasing spread of latent TB infection. If many people still haven't heard about it, then how can we explain it better to them? What can we do? Can someone enlighten them through initiatives?**

Patients with increased faith in superstition often believe in the speeches of people from lower castes as well. There are many such individuals in society, such as significant ascetics, saints, Bhuvaji, Morari Bapu, and Babaji. If these individuals spread awareness about TB in their speeches and these messages are accepted quickly by society, they are readily accepted.

**What steps do you believe the National TB Elimination Program should take to specifically address the latent TB concerns among agate workers in Khambhat? Are there specific policy changes or resource allocations that could facilitate these steps?**

We are often forcefully told by the NTEP authorities that we have to implement certain strategies. We have developed a formula within the NTEP program. Whatever entries we make; the target is set. Within the target, changes in the program are made every four months. If we explain to the health workers that a certain task should be completed in two months, another new implementation may occur in the meantime. My point is that whatever implementation is required, it should be done systematically so that your health workers can understand that this is part of your TB program. If you keep changing things every day, the burden increases, and the work related to TB that we do in the field is affected.

We don't always have time to visit patients in the field. Sometimes, we have to focus on data entry, and sometimes we have to work on the computer. It's not like we can smoothly transition between these two tasks. If there is any interruption, then one group of people should focus on systematic work in the field, and another group should focus on computer work. If someone who usually works in the field is assigned computer work, they may not pay attention to the field. There may be obstacles in work. After that, mistakes may happen, which should not be forced in true reporting.

**Is there anything else you would like to say that you feel you were not able to say during the interview?**

No.

**In-depth Interview 4: Community health officer (TB health worker Khambhat), 5 years of experience**

**What are your perceptions regarding the significantly higher prevalence (58%) of latent TB infection among agate workers compared to the general population (31%)? What explains the near doubling of LTBI in this group?**

Here, everyone is supportive in their work, even if it's not the responsibility of the local governing body or our ASHA workers. Together, we organize awareness campaigns in the village. Our goal is to make India TB-free, so we have various programs for that. After identifying a TB patient, known as the Index Patient or TPT patient, they undergo treatment until completion of the entire course. We also ensure follow-up for both the index patient and their contacts (IP-CP) to ensure they complete their treatment. Actually, one supportive reason for this could be that those who complete our treatment and eventually test negative contribute to this high positivity rate.

**So, it seems like you feel that the increased positivity in the IGRA test results, with 269 out of 463 individuals testing positive, indicates a rise in latent tuberculosis infection (LTBI) among them due to their line of work. What is your opinion on their occupation playing a role here?**

Here, more people work in environments with silica dust exposure, particularly the employees being tested, leading to an increased spread of infection. Therefore, the positivity rates have risen predominantly due to this factor.

**Could you elaborate on the challenges faced in conducting LTBI testing among agate workers, considering factors such as the high cost of kits and the need for sophisticated laboratories?**

There aren't any major challenges as such, but sometimes patients may not be convinced to undergo testing. Otherwise, there aren't any issues during follow-up, and all procedures such as X-rays and sputum tests are carried out smoothly here.

**Are there lessons from the TPT program for household contacts (currently ongoing program being managed by Alert India NGO) that can be applied to agate workers?**

Yes, that's a good point. Currently, the TPT program is running well here in Shakkarpur, meaning that if we initiate the TPT program when a TB case comes up here, there will be fewer chances of TB cases emerging in the future.

**Based on your experience, what can we do to reduce the burden of latent TB infection (LTBI) among our workers undergoing IGRA testing?**

To reduce it, here in Shakkarpur village, we have Information, Education, and Communication (IEC) materials that explain how TB spreads, why it's important for contacts of positive patients to take less medication, and how having low immunity can lead to developing TB. Therefore, we need to conduct IEC activities and make efforts to reduce infection among the population by providing them with information. We should also provide them with pyridoxine tablets.

**Currently, when you start the TPT program for household contacts, how has been the response from these individuals? Have there been any complaints or adverse drug reactions so far? How is their support system?**

Currently, about 60-70 people in our village are part of the TPT program, and there haven't been any complaints or reports of adverse drug reactions among them. There have been no negative results.

**What is the awareness level of these individuals? Since they may not have any symptoms, and their X-rays may be normal, and we tell them they need to take medication for 6 months or 3 months, what is their awareness like?**

We explain to these individuals that if they take medication for 6 months, the chances of them getting TB will be reduced, and there won't be any second-time chances. We reassure them that there won't be any problems later.

**We've discussed that currently, when the TPT program is initiated for household contacts, we rule out symptoms and X-rays, and we don't conduct IGRA testing. Instead, we rule out these two factors and directly provide them with TPT. Since IGRA testing is quite difficult among our workers, can we use the same strategy here by screening for symptoms and ruling out X-rays, and then directly providing them with TPT?**

Yes, currently, in the TPT program, we start it when sputum is negative and X-rays are also negative, so we can start TPT directly afterward.

**What steps do you believe the National TB Elimination Program should take to specifically address the latent TB concerns among agate workers in Khambhat? Are there specific policy changes or resource allocations that could facilitate these steps?**

Actually, currently, sputum testing is being done, but when we refer patients for X-rays, it creates problems for these individuals. Otherwise, there are no other issues.

**Is there anything else you would like to say that you feel you were not able to say during the interview?**

Actually, there is an increasing number of TB cases here, so it's also challenging to work within it. I think what should be done for these individuals, even the staff can get positive, so what should be done for these individuals, so that they don't face any problems afterward. Currently, we have 8 ASHA workers who are also working in this, if their immunity decreases, they can also get infected.

**So, what can the government do if even ASHA workers can get infected? If you have any suggestions, you can share them?**

If the government supports us at the sub-center level, it won't be a problem.

**In-depth interview 5: District lead TPT program, 7 years of experience**

**What are your perceptions regarding the significantly higher prevalence (58%) of latent TB infection among agate workers compared to the general population (31%)? What explains the near doubling of LTBI in this group?**

These are people who live in slum areas. And when we go to meet these people, there is no awareness of how TB spreads or how we should take precautions in our project. When we meet the households of patients, these people have no idea how TB spreads or how they should take precautions. They need to know how TB spreads and how they can keep themselves safe from it. It can spread through the air, and attention should be paid to where precautions should be taken. These people generally work in areas where there is dust, so they are generally living with others in those areas. So, this is also a reason why the prevalence ratio is higher among them.

**Do you think there could be a difference between working with agate polishing and working with agate stone itself, in terms of the impact on health?**

Yes, there could be a difference. People who work with agate stone might be exposed to dust in the air, which could affect their lungs and immune system, leading to a higher prevalence ratio among them.

**In your opinion, what factors contribute to the high positivity of latent TB infection among agate workers, especially those engaged in high silica dust work like polishing or chipping (2 times higher) than drilling (65% vs. 52%)?**

Yes, there could be one thing: those who are polishers work with machines, which could create more dust in the air, leading to higher chances of respiratory issues. So, if most of the work involves using machines, that could be a reason. However, I'm not exactly sure; this could be one of the reasons.

**Could you elaborate on the challenges faced in conducting LTBI testing among agate workers, considering factors such as the high cost of kits and the need for sophisticated laboratories?**

When it comes to IGRA testing, initially, we faced challenges in getting patients to provide blood samples because there was a lack of awareness about the test and some resistance from patients. Also, the test is costly, and the government may not provide the facility for it, which can be another challenge. Moreover, some patients may not be able to afford the test even if they want to. So, one solution could be to use alternative tests like the Cy-Tb test, which has been launched by the Government of India. If it proves to be as efficient and effective as IGRA testing, then we can use it as an alternative.

**You have been involved in the TPT program for household contacts for quite some time. Are there lessons from the TPT program for household contacts (currently ongoing program being managed by Alert India NGO) that can be applied to agate workers?**

When it comes to the availability of drugs, I can say that until now, the availability of drugs has been good under the Government of Gujarat. In our project, almost all eligible individuals received TPT. However, there is currently a slight shortage. In such cases, the Government of India can focus more on TPT under the National Tuberculosis Elimination Program (NTEP). If the budget for TPT is increased, it would be beneficial. Additionally, if a company agrees to donate funds specifically for this cause through Corporate Social Responsibility (CSR) funding, we can also utilize that to help ensure availability.

**Based on your experience and lessons learned so far with the TPT Program and Household Contacts, how can we effectively utilize it when implementing the TPT Program among agate workers?**

In terms of my major experience with TPT, I have been working on it for the last two years. Generally, we provide TPT to household contacts, who are usually healthy individuals. Sometimes, there is resistance from them because they don't see the need for testing or taking medicine. So, strong counseling is crucial. When we initiate TPT for someone, if the initial counseling is strong, they are more likely to complete the course. Otherwise, they may drop out in between. Therefore, we focus on strengthening counseling, with the help of our staff. When doctors diagnose and counsel patients, if they add a point about household TPT during follow-ups within the general health system, it can benefit both the patient and the household members by helping them understand the importance of latent TB treatment. So, efforts from both the health system and the doctor's end are necessary. This can lead to benefits in diagnosing and treating latent TB infection.

**In your opinion, if we want to reduce the burden of latent TB infection among agate workers, what potential solutions should we implement?**

If we want to reduce the burden of latent TB infection among agate workers, we need to target this particular population. Initially, we should conduct tests for latent TB infection among them, and once the results are available, provide treatment promptly. If we can diagnose them early and provide timely treatment, we can control the spread of infection and prevent further transmission. So, the main focus should be on early diagnosis and timely treatment.

**What steps do you believe the National TB Elimination Program should take to specifically address the latent TB concerns among agate workers in Khambhat? Are there specific policy changes or resource allocations that could facilitate these steps?**

Yes, when it comes to the population of agate workers, we need to create a monitoring system for those areas where the population of these individuals is higher. Similar to Active Case Finding (ACF) activities conducted to diagnose TB, we should establish a system where routine diagnostic tests are conducted or eligible individuals are provided with treatment. For effective implementation, we need to allocate tasks, build capacity for counseling, and provide training for the entire process of the PMTPT program. Without proper training, we won't achieve satisfactory outcomes.

**Is there anything else you would like to say that you feel you were not able to say during the interview?**

Surveys indicate that latent TB infection affects 40-50% of the population in India. Therefore, targeting this population would be significantly beneficial, especially considering the increased risk of active TB among them. Creating specific activities within our general health system for these targeted populations can help us effectively prevent the spread of TB.

**In-depth interview 6: State-level official (NTEP), 2 years of experience**

**What are your perceptions regarding the significantly higher prevalence (58%) of latent TB infection among agate workers compared to the general population (31%)? What explains the near doubling of LTBI in this group?**

According to me, there could be 2-3 reasons. Firstly, the patient may have been exposed to dust, which could possibly lead to a decrease in their lung's clearing capacity, thereby potentially causing infection due to exposure to dust or possibly due to their low immunity. Another aspect to consider is the nutrition status of such individuals, especially in workers like these, where we should assess their nutritional status at least once, and linking it with BMI might give us a rough estimate of their actual nutrition status. Another factor could be their socio-economic condition, as it could be a compounding factor. It could be possible that they are working in environments where ventilation is poor or their beliefs and understanding might not directly contribute to infection but could affect their living conditions, which in turn could affect their infection.

**Could you elaborate on the challenges faced in conducting LTBI testing among agate workers, considering factors such as the high cost of kits and the need for sophisticated laboratories?**

Firstly, the challenge lies in the availability of IGRA testing itself, as there is almost nil availability of IGRA testing in government setups, and it's rare even in private setups to find a laboratory capable of conducting IGRA testing. If we intend to continue with IGRA testing, as it is ELISA based, we can increase the capacity by providing training to microbiology departments in medical colleges. Secondly, if we need the kits, the government can conduct common tendering and procure the kits, thereby increasing accessibility district-wise. Another point to consider is that the Government of India has conducted the Cy-Tb study, and if the results are positive and procurement is underway, then IGRA testing might not be necessary as the sensitivity and specificity of Cy-Tb are good. Cy-Tb could be a better option in the field, and it might not require much technical expertise.

**What challenges do program functionaries face in providing Tuberculosis Preventive Treatment to agate workers, especially in terms of the availability of Isoniazid and combination drugs like Isoniazid-Rifapentin?**

The issue with the availability of TPT has been faced recently because for the past 1-1.5 years, there has been a delay in its procurement due to the delay in the procurement of Isoniazid, which is generally used as TPT. This delay has occurred due to various reasons, including delays in central procurement of TB drugs and states not being ready to procure TPT as soon as it is available. However, considering the high prevalence of infection in populations exposed to silica dust, and given the challenges with IGRA, we can reduce this shortage by considering two strategies. Firstly, if the rate contract for TPT becomes available, it can be included in the Essential Drug list and procured at the state level. Moreover, when there is a shortage of Isoniazid, the district TB officer can procure it locally based on the rate contract. Secondly, in the near future, if the Central TB Division procures a significant amount of TPT, the situation might not remain as critical as it is now.

**Are there lessons from the TPT program for household contacts (currently ongoing program being managed by Alert India NGO) that can be applied to agate workers?**

The issue lies with household contacts. We generally do not provide TPT directly without testing below 5 years old, but for those above 5 years, we do provide TPT. However, if a study reveals that more than 50% of vulnerable groups are infected, it might not be incorrect to provide TPT directly without testing. There isn't a clear guideline in place for this vulnerable group, but it depends on the state to decide whether to test and treat or only treat. If the state decides to follow the "Test and Treat" or "Treat Only" model mentioned in the TPT guidelines, then there shouldn't be an issue with providing TPT without testing.

**According to our PMTPT guidelines, silicosis is considered as a high-risk group. However, diagnosing it is challenging as physicians may not readily suspect it, and there is a lack of training for diagnosing it. In our study, we found a 58% prevalence of infection among the silica dust-exposed population, but when we look at the Khambhat population, where people mainly use the water grip method, dust exposure is significantly lower. Considering that approximately 52 million people are exposed to silica dust nationwide, what is your opinion on silica-dust-exposed individuals as a high-risk group under the NTEP-PMTPT program?**

Currently, the high-risk group we consider is evidence-based, relying on studies conducted in certain areas. The addition of high-risk groups is based on evidence from studies. Therefore, the evidence we have generated from the study, showing a high prevalence of infection among those exposed to dust, can potentially be used to include them as a high-risk group. Additionally, if we conduct further studies or meta-analyses that consistently show a high prevalence of infection among silica dust-exposed populations, this evidence could justify the expansion of the program at the pan-India level to cover a larger pool of individuals.

**What are the potential solutions to reduce the burden of LTBI among agate workers?**

Regarding dust exposure, we need to consider measures to mitigate the reasons behind it. For instance, the use of personal protective equipment such as masks when cutting or polishing, or the use of water during such activities to reduce dust generation can be helpful. Secondly, workplace policies can be implemented, including screening for dust exposure at the time of employment and regular symptomatic screening. Additionally, for high-risk groups where testing is available, BMI screening can be conducted, and if undernourishment is detected, general counseling for diet improvement can be provided. While directly increasing BMI may not be possible in the program, at least diet counseling or any feasible interventions could be incorporated into the program.

**Is there anything else you would like to say that you feel you were not able to say during the interview?**

No.

**In-depth interview 7: Medical Officer, Anand district (13 years of experience)**

**What are your perceptions regarding the significantly higher prevalence (58%) of latent TB infection among agate workers compared to the general population (31%)? What explains the near doubling of LTBI in this group?**

As you mentioned, we all know that the prevalence of TB infection in the general population is approximately 30 to 35%, and among Agate Workers, due to Dust Exposure and lower immunity, the possibilities of TB Infection might double there. We are aware that Key Populations, such as those with Diabetes, HIV, Cancer, Dialysis patients, those on Immunosuppressive Therapy, and those associated with occupational hazards, have almost double the chances of contracting TB infection. Therefore, perhaps Dust Exposure becomes such a factor that, over a long period, say 5, 10, or 15 years of working as agate workers, the rate of infection significantly increases. As you represent in your report, almost double the chances of infection are present there. Hence, it seems to me that prolonged exposure to dust and lowered immunity may lead to increased infection rates among agate workers compared to the general population.

**In your opinion, what factors contribute to the high positivity of latent TB infection among agate workers, especially those engaged in high silica dust work like polishing or chipping (2 times higher) than drilling (65% vs. 52%)?**

We all know that many Agate Workers used to work without water drips previously, which means they work in an atmosphere where dust is more prevalent. After that, policies were made that all these machines should be shut down, and machines with water drips built-in should be used. So, when Agate workers work with these machines, especially in Polishing, it is observed that those who use machines with water built-in, the chances of dust in the atmosphere are significantly reduced. Thus, we may see that in those who work in Polishing, using machines with water inbuilt, the atmosphere's dust level is significantly lower. Consequently, we need to pay more attention to those who do this job using Water Inbuilt machines. It might be possible that this could be a major reason. In cases where the workers do not use Water Inbuilt machines, we must see that drilling in the agate stones may become the means to produce dust. Thus, even though they do not use Water Inbuilt machines, but they use some instruments to make small holes in stones for drilling work, we need to pay more attention to them. And if those who use Water Inbuilt machines or those who do the same job with one particular task, their exposure to dust is reduced, so maybe there are fewer chances of infection in them. So, although we observe a reduced rate of dust exposure and perhaps lower TB prevalence due to reduced dust exposure in the atmosphere, it might still lead to more chances of infection over a prolonged period for those individuals who are constantly exposed to increased dust exposure due to drillings while working. Therefore, I think this could be a reason that with increased dust exposure, even though the prevalence of TB is lower due to reduced dust exposure, individuals who are continuously exposed to increased dust exposure might still have double the chances of infection in them.

**Could you elaborate on the challenges faced in conducting LTBI testing among agate workers, considering factors such as the high cost of kits and the need for sophisticated laboratories?**

When it comes to IGRA testing, the cost for conducting one test ranges from 1800 to 2000, and even in this, the tests we conduct at the field level require us to work with blood samples. These samples need to be maintained with cold chain maintenance until they reach the designated laboratory, where testing can be done. This process usually takes almost 2 to 3 days, which means 48 to 72 hours until we receive the report. All these hurdles are associated with it. Another test that can be considered is the Mantoux Test. While this test can be conducted on-site, if we need to check for specificity or sensitivity between the two tests, IGRA testing might be more sensitive than the Mantoux Test. Moreover, when we talk about government interventions, the government currently allows both the Mantoux test and IGRA test for infection testing. But implementation for other tests meant for infection has not yet occurred in the program. So, concerning the NTEP program, the government should consider whether tests like Mantoux that involve less cost and more individuals with dust exposure can be included. By implementing new tests under the NTEP program, we can identify infections earlier, and those identified with infection can also be given TB Preventive Therapy (TPT). This can significantly reduce the chances of TB disease in these individuals and provide relief from morbidity.

**Are there lessons from the TPT program for household contacts (currently ongoing program being managed by Alert India NGO) that can be applied to agate workers?**

When discussing the NTEP Program, priority individuals for TPT are those who are HIV positive, on ART, as they have increased chances of TB infection. So, the program has been implemented for them, irrespective of the test. Another most important point is that individuals who are patients of TB, identified as index patients, and their contacts are residing, irrespective of the test, are provided TPT according to the guidelines. Initially, when we discussed the RNTCP, we used to provide TPT to children under 5 years old. Now, we are providing TPT irrespective of the test to all household contacts. So, in these two cases, contacts of TB patients, where TB disease's chances are higher, and another PLHIV person, where the chances of TB disease are increased, TPT is being provided. Similarly, our third objective is to provide TPT to high-risk groups such as those with silicosis, undergoing dialysis, cancer patients, and those on immuno-suppressive therapy. TPT is being discussed for all these individuals. However, based on your study, the report I have seen, infection rates have doubled in individuals associated with polishing and chipping and are slightly lower in individuals associated with drilling. Therefore, the guidelines might need to be modified in cases where TPT is provided irrespective of the test for individuals with silicosis. Instead, in the place of silicosis, where dust exposure is high, and where individuals have been working for more than 5 years, if TPT is provided irrespective of the test, we can reduce the chances of infection turning into disease. And if we start TPT early, even if we rule out active TB, these individuals can possibly live a good life without morbidity.

**What are the potential solutions to reduce the burden of LTBI among agate workers?**

First, it is necessary to conduct integrated work. Along with the Health Department, the Industrial Department should also be involved, and individuals working from home, such as agate workers, should be registered. Based on our studies, individuals engaged in activities like polishing, chipping, and drilling can be offered alternative job opportunities to reduce their exposure to dust. If such opportunities are not available, then creating zones where machinery with inbuilt water systems can be installed could significantly reduce the chances of dust exposure in the atmosphere. It is essential that the government pays attention to this initiative from the beginning, ensuring that new machinery with water inbuilt systems is introduced to minimize dust exposure among agate workers. Moreover, all agate workers and individuals exposed to dust, whether in Gujarat, specifically in Khambhat and Morbi, or in Rajasthan, where different industries with increased dust exposure exist, should undergo testing. If the cost of IGRA testing, which is around 1800-2000, is borne by the government, then perhaps a new test can be implemented, leading to an increase in the number of tests conducted under the program. By identifying individuals with double the number of infections, as indicated by our study, we can intervene more effectively. If TPT, TB preventive therapy, is made freely available by the government, then timely administration of TPT to those identified can lead to a reduction in the incidence of disease conversion among infected patients.

**What challenges do program functionaries face in providing Tuberculosis Preventive Treatment to agate workers, especially in terms of the availability of Isoniazid and combination drugs like Isoniazid-Rifapentin?**

Currently, our study primarily focuses on Khambhat, and according to our findings, there may be a slight increase in the prevalence of infection among individuals engaged in polishing, chipping, and drilling activities over the past five years. If 60% or more of individuals in the program are found to have infection rates above 35%, the government needs to pay special attention because if such high infection rates are found, and if INH TPT is to be administered for up to six months, then one person will require six months of INH treatment free of cost. Hence, it is essential to maintain uninterrupted stock supply and develop a calculation strategy to ensure that all medicines received by the government from the industry and covered under MoUs signed annually by the government are made available regularly without interruption at every PHC level or Health and Wellness Centre.

**How can we do awareness generation in a better way so that mass awareness can come?**

We educate individuals who have been exposed to Dust Exposure, such as Agate workers, about health. After providing health education, awareness is a separate matter. After awareness, it is about implementing it. These people understand and consider implementation after being made aware. Stigma remains, whether one is literate or illiterate, it still exists to some extent. Stigma doesn't disappear instantly; it diminishes gradually. I'm not saying it remains 100%, but the extent of stigma still varies from person to person. If someone has a cough and is advised to undergo a chest X-ray, he/she may not be ready. Thus, two things can happen here: one, we educate based on our experience in the field, and two, what guidelines exist in the industry. If the government or private sector issues guidelines, they should be registered, and they should be told whether to use this machine or to work at a certain place. If these people receive regular health check-ups twice a year, they will be safe. To prevent Dust Exposure, they should wear goggles, masks, hand gloves, and aprons. We often talk about these things, but perhaps they fear that there are no registered guidelines in the industrial guidelines and, thus, have some fear daily. Perhaps it is because the earnings are low, or there may be a poor situation. They hesitate to go to government or private setups for fear that something might happen. Awareness also involves stigma. And if someone suffers some discomfort on a daily basis, it may not come to the forefront for immediate treatment. I feel that in order to work safely, they should be provided a place and machinery, and if they are provided with this twice a year for health check-ups, they will be less afraid over time. And if they see any sign symptoms, they will come forward for immediate examination and treatment, which is what I feel.

**What steps do you believe the National TB Elimination Program should take to specifically address the latent TB concerns among agate workers in Khambhat? Are there specific policy changes or resource allocations that could facilitate these steps?**

As far as I have been associated with the Khambhat area for the last 13 years with Agate Workers, initially, Dr. David used to work in the Khambhat TB Clinic, and he did very good work there. Living with agate workers and providing them with regular health check-ups and IEC, we used to make efforts to reduce stigma. So, what you are saying, in this, from the government side, not just the Central TB Division, but the industrial zone in this, also becomes active, we can reduce the incidence in all of this. Another thing is that often, the term "Silicosis" has been used less, and the term "Dust Exposure" or wherever dust emanates, if this term is used, then all individuals affected by it, many more populations, which are linked with different occupations, can be included. So, if we test everyone where Dust Exposure occurs, and check the infection in everyone, and detect infection and give TB preventive treatment, then the incidence of the disease can be reduced…. TB incidence which was previously reduced by around 2% per year, which is around 10-15% currently… I believe we can bring in a reduction of 25% every year in TB incidence. It is very important to pay attention to the high-risk group in our NTEP guidelines… if we pay maximum attention to this high-risk group, then we can reduce the incidence of infection and TB significantly, in my opinion.

**Is there anything else you would like to say that you feel you were not able to say during the interview?**

We have discussed everything, but, since I am a person associated with health, I may have less detail about administration, but if we integrate different departments into this, then we can consider that if we can reduce the infection in individuals affected by Dust Exposure. If integration happens, then we may get many more positive results in this. We make efforts to reduce stigma after the problem arises, we make efforts for testing as well, but there are many difficulties in this too, because even testing sometimes has a fear, that maybe after being diagnosed with Silicosis, after being diagnosed with TB, their job will be lost. So, even in testing, many people face a lot of difficulties. Even when treatment is initiated, then there may be hesitations in people, which may be only 1 or 2%, but we observe this among Agate Workers or Silicosis patients. I believe that even after all this, we make many efforts, but if this happens or not, then if we try to do this with the department of others, then maybe we can get better results.

**In-depth interview 8: State lead TPT program, 1 year of experience**

**What are your perceptions regarding the significantly higher prevalence (58%) of latent TB infection among agate workers compared to the general population (31%)? What explains the near doubling of LTBI in this group? What factors contribute to the high positivity of latent TB infection among agate workers, especially those engaged in high silica dust work like polishing or grinding (2 times higher) than micro-perforation (64% vs. 51%)?**

Thank you, so as we know the greater the dust particles, there is higher chances of them being lodged in the lungs and the alveoli and if there are smaller dust particles, there is less chance of them being stuck in the alveoli, so this might lead to the 10%-13% difference you are seeing in terms of the same. Because polishing might lead to smaller particles compared to chipping, so that's one of the reasons. Again, we need to see more data but generally chipping which is a work which needs heavy manpower, means more power, so it's generally being done by younger population. So we need to also maybe correlate the risk of tobacco smoking with the same. And polishing is generally done in a resting position from what we discussed, there also the respiratory patterns and the personal safety equipment might also play a role in the difference. Summarizing since both are different nature of work, so the manner in which the work is being done might play a major role in the same.

**Could you elaborate on the challenges faced in conducting LTBI testing among agate workers, considering factors such as the high cost of kits and the need for sophisticated laboratories?**

Yes, so regarding the challenges you told, these are universal challenges being faced by every functionary working with the NTP or any partner organization in the TPT sector, but recently the central TB division have introduced Cy-Tb test which is relatively cost effective test compared to IGRA or any other test which can be used to screen out for latent TB infection, so maybe in the near future once the availability of the kits get secured, maybe we can shift to Cy-Tb instead of the costly IGRA to mitigate the cost factor of the kit.

**What challenges do program functionaries face in providing Tuberculosis Preventive Treatment to agate workers, especially in terms of the availability of Isoniazid and combination drugs like Isoniazid-Rifapentin?**

Okay, so first of all because of the strong coordination by the state governments, till date Gujarat has not faced any major challenges in terms of availability of isoniazid or 3HP isoniazid-rifapentin combination. But yes because of the what we say unpredictable demand, there might be challenges in the near future in terms of availability. So pursuant to the same I think that advanced order should be placed, projections should be made and proper cycle should be established in terms of drug dispensation. So that the drug gets drug procured with lead time of at least 2 months, so that the treatment gets continued. But other than the drug availability, I think one major factor is the counselling skills of the people who are introducing the TPTs. Since we are offering the treatment to the patients or I would say to the people who per-se do not have any active infection, for that they might be asymptomatic or they have mild symptoms but they do not have any active TB. So convincing them to take TB preventive therapy might be a challenge, more significant than the availability of drug. So for that along with drug we need a proper manpower trained with counselling skills to explain the need and importance of the TB preventive therapy for the agate workers.

**You said that there is an issue of availability of the TPT drugs, so what exactly, can you elaborate what exactly is the issue, is it the manufacturing, is it the procuring, is it the import of drugs, where exactly is the problem?**

So based on my… as per my perception, the issue per-se is being with the manufacturing, because the demand is there, procurement process is there, but there are not enough manufacturers who can manufacture in bulk the quantities required to supply TPT to whole country like India. Just for example, last year India had around 28 lakh TB cases, of which just let's say 60% of pulmonary cases also goes to around 15-16 lakh pulmonary TB cases, multiplying by 4 household contacts, we would need around 64 lakh doses of TB preventive therapies. Currently no major pharma company is working on, so there are just a couple of pharma companies working on it, so obviously their manufacturing capacity is far less than the demand, so if something is done in that, then we can ensure the, and that the TB preventive therapy is in stock.

**What are the potential solutions to reduce the burden of LTBI among agate workers?**

One of the solutions is routine occupational health screening of the workers, setting up a dedicated occupational health clinic or a centre where there are high concentration of agate workers… so, under the supervision of occupational health specialist, maybe proper practices can be taught to the workers of how to protect themselves from silica dust exposure or agate exposure… like wearing of proper personal protective equipment, routine screening, routine check-up, when to visit a doctor, if they are being taught to the workers, then their chance of exposure decreases. Second, awareness among the local population regarding TB and TB infection. And the third, I would say a radical solution would be, since you found around 60 to 65% prevalence among them, maybe based on the population, all can be given TPT treatment once… though there is no documented evidence, but maybe you can have a study where you give TB preventive treatment to all of the 5000 people, minus their exclusion criteria of active TB, and see let's say after 6 months or 1 year, the prevalence of TB, I mean TB infection, so it can be one of the thing.

**You have been involved in the TPT program for household contacts for quite some time. Are there lessons from the TPT program for household contacts (currently ongoing program being managed by Alert India NGO) that can be applied to agate workers?**

So one of the major learnings has been that initiating in the first follow-up, they are the most important things in terms of TPT. So not only initiating, if the agate workers who have been started on TPT… continues for the first 1 month, as you are going for 3HP, and that is, continue for the first 4 dosages, then it's more likely that they will complete the treatment. So proper follow-up and support is required in the first month of treatment because that's the time period when ADR are more likely to occur, loss to follow-ups are more likely to occur and after a month, the routine is being set and the end seems closer to the person regarding the therapy. So that's one of the key takeaways is that once you start the therapy, post proper counselling, ensure follow-up for the first 4-5 weeks which will improve your outcome significantly.

**Any specific suggestions as far as the ADRs are concerned? Since you mentioned that there are more ADRs with 3HP as compared to 6H, any specific suggestions which we can implement?**

First and foremost is the field workers who are initiating the TPT, they should be properly equipped about the knowledge of ADRs and which are the mild ADRs and which are more likely to occur but which are not harmful like discoloration of urine, so it's one of the common. So upfront they need to be honest with the worker and they need to tell them the ADRs which are likely to occur and set the expectations that yes, this ADR is bound to occur but you need not worry. So that the psychological effect of the ADR is decreased. The second thing is the timings should be such that one of the common ADRs is weakness. So maybe if you give it on the day of the weekly off, then they will have more time to rest and recover it for the ADR of weakness. Then proper medical advice should be sought and the workers who go and start the TPT, they should be equipped to identify danger signs, where they should refer the person to doctors or medical professionals whenever the situation seems worsening. Also one of the common ADRs is acidity, so that should also be explained to the patient in advance so that loss to follow-up does not occur.

**What steps do you believe the National TB Elimination Program should take to specifically address the latent TB concerns among agate workers in Khambhat? Are there specific policy changes or resource allocations that could facilitate these steps?**

Okay, so first of all the PMTP guidelines of NTEP currently does not allow for giving TPT to agate workers or high-risk populations other than HIV, the immunocompromised and household contacts. Silicosis being a high-risk condition, there should be a policy change that all the high-risk workers other than those mentioned in terms of contacts should also be provided TPT to reduce the prevalence of TB infection and second point as I mentioned earlier, occupational health clinic should be set up or awareness should be set up so that workplace infection, cross infections or increasing risk factors does not occur.

**Is there anything else you would like to say that you feel you were not able to say during the interview?**

No, I guess we covered most of the points in the interview and I am really interested in looking forward to the findings of your research. Best of luck and thank you.

**In-depth interview 9: State lead new initiatives (NTEP including TPT program), 9 years of experience**

**What are your perceptions regarding the significantly higher prevalence (58%) of latent TB infection among agate workers compared to the general population (31%)? What explains the near doubling of LTBI in this group?**

Almost all the trials or the earliest studies also suggest that this prevalence will be higher in the agate workers or who are having some silica exposure. Some TB prevalence survey also showed in many states it was conducted, and in Gujarat, we can see the areas have been selected where this kind of occupations are carried out by the people. So those kind of people are having the high prevalence compared to other community members so definitely this seems to be increase. This is also needs to be analysed with the previous exposure of TB or the workplace where the agate worker is working in the industry or the company where the other people’s workplace… the atmosphere… everything matter a lot. But definitely its seems to be incremental than the other community people.

**In your opinion, what factors contribute to the high positivity of latent TB infection among agate workers, especially those engaged in high silica dust work like polishing or chipping (2 times higher) than drilling (65% vs. 52%)?**

Yes, definitely chipping actually having a more exposure of silica dust than the polishing part but the exposure of silica dust along with the agate workers mainly what we have seen in the state or some of the district where they are working… their nutrition… mainly are malnourished also. So dust exposure which is higher in this point of agate work and when they do drilling and all the component will be in the air for some time and they are having a higher exposure with their malnourished status and their living conditions. So I guess all the 3 to 4 parameters which is majorly depending on the latent TB exposure needs to be considered and that is why it is higher in the polishing part because polishing part is normally just sitting and doing polishing where higher amount of exposure will be there so definitely its higher in such work.

**Could you elaborate on the challenges faced in conducting LTBI testing among agate workers, considering factors such as the high cost of kits and the need for sophisticated laboratories?**

Based on my experience conducting any test for any people like TB test or Latent TB test or for any test… so these are the special groups where we are just wanted to check their positivity or the prevalence of some of the disease but getting the testing done for normal person who is not having any of the symptoms for anything it is little tough. So the first point is that counselling is required. Why is this testing to be done and post-testing what could be the conditions where they are required to be on medication or not. So that counselling needs to be done at the first point. The second thing is whenever we ask any patient to be done the test… the fear zone they come across so because of the fear factor they refuse to do some testing and because of that only the high level of counselling needs to be done for the agate workers. And the local person or some CHC/PHC staff or some government staff can help us to do good amount of testing, ASHA workers also can be a very helpful in such case where they convince this kind of workers to do some testing and spreading awareness is the major point where we can convince them for at least testing which kind of testing we are going to do. So for Latent TB infection, if we are testing them so TST, IGRA and now Cy-Tb are available in the market. So easy test and easy to handle and fastest result providing and the accurate result providing test should be preferred so IGRA and Cy-Tb needs to be done and based on that at least we can have result in our hand which is reliable that the patient needs to be considered as a Latent TB infection individual.

**You mentioned the Cy-Tb so probably you can focus more on or elaborate more on how Cy-Tb can overcome these challenges. What is the turnaround time of a testing through Cy-Tb as compared to IGRA?**

This is great that you considered IGRA for the agate workers and because right now that is the only test which can give the accurate latent TB infection level of the individual patient or the individual households so that is the greatest. Cy-Tb is just coming up and like it is just under some trials and now it is going to be introduced in the country soon. So, Cy-Tb is having a similar efficacy like IGRA and it is easy to do the test and any of the field workers can do it with the proper training they can perform this test at patients’ houses also as well as in CHC also. The cost effectiveness is in IGRA… it is costing higher but in Cy-Tb costing is like TST and efficacy is of like IGRA. So it is providing both of these things. So, cost effectiveness is also less and accuracy of result is high. In 1 or 2 days you can have a ready-made result which is like a prompt result like IGRA as per the efficacy. So Cy-Tb is the future, with Cy-Tb it will be easy to screen more people within the community.

**You have been involved in the TPT program for household contacts for quite some time. Are there lessons from the TPT program for household contacts (currently ongoing program being managed by Alert India NGO) that can be applied to agate workers?**

TPT initiation is not a bigger challenge if you have a counselled individual in the beginning itself, while convincing them for the testing part, once the test is available. And, if the patient or the household is aware that if the test comes positive they have to be on treatment for prevention of TB… so that is one part. The current challenges on the treatment part like drug availability in the country so based on the Gujarat government support and all the individuals or all the officials they have predicted well. So as of now we are not facing any challenges in drug initiation like we have both the regimen available in the state - 3HP as well as 6H - so few of the districts are under 6H and many of the districts are under 3HP regimen. So, the major challenge with the TPT initiation what we found is 6H per say because 6 months without any disease if you have to be on treatment if I personally also need to consider, I will also think twice or thrice before initiating treatment and they are the normal individual so they do not understand why this 6 month treatment is required without any symptoms or signs. So, that is the major challenge and we observe also the patients who are on 6H they are not continued on the treatment they have left the treatment in between… 3 to 4 months after the treatment initiation after they have left they found LFU because of 6 month of longer duration treatment. Similarly, 3HP is weekly regimen and 3HP sounds better… the result also good and due to weekly regimen for the 12 doses only… if we convince the individual they are ready to take that regimen. The major challenge with the 3HP what we have found is INH dose of 900 mg normally in a single shot, so that some complications the patients are observing, that is the only challenge we are currently facing with 3HP. Rest, drug-related challenges we are not seeing much in the initiation part because the patients or the households are already ready like we are having an experience of index TB patient household contacts where we are providing the TPT and when the one of the TB patient in your house who is currently suffering with the TB. So the fear which is coming in their mind of the households and with the proper counselling these parts playing a major role and with that only they are ready to go on treatment. So a good number of patients have completed their 6H treatment of the 6 months so that is the good part for the regimen available.

**What challenges do program functionaries face in providing Tuberculosis Preventive Treatment to agate workers, especially in terms of the availability of Isoniazid and combination drugs like Isoniazid-Rifapentin?**

Yes, in country there is a drug shortage of INH and 3HP also because there are two reasons - government has predicted like somewhere around 65 to 70% of the total households who will be on TPT… that estimate got higher might be the reason because the lack of testing of IGRA. Based on IGRA test, you can get around 30 to 40-50% positivity. Ideally that 40-50% patient should be on TPT but in many districts or the states without testing of IGRA, X-ray screening has been done to rule out active TB, and then TPT has been initiated. So in that case around 80% of household became eligible for TPT initiation. So may be that prediction was not predicted at that point of time may be the reason for shortage of INH. The 3HP, government has tried a lot since the last 6 to 8 months, to get on board, but manufacturers were not available or manufacturers were getting ready and now things have moved. So I guess in a short time we will get 3HP across the country, that’s what my understanding is.

**What are the potential solutions to reduce the burden of LTBI among agate workers?**

Solutions like government is well aware they are doing their major steps like providing all the solutions… so major thing what I think is for agate workers where special screening on every six months at least twice a year… we can screen such occupational group not only the agate workers but the diamond industry, the people who working in that, some cloth industries in Surat or some other cities where they are working. So this kind of people needs to be screened at least twice a year. And not to be run as a major programme, but yes, if government is well aware that Khambhat district is having a good numbers of agate workers, so Khambhat UHC/CHC or some general health system needs to be involved for such screening and post-screening if they found some high risk pattern they need to be initiated on treatment. So that is the major thing we can do specifically for this group.

**When we say initiating the treatment after screening so again it would require IGRA testing or may be Cy-Tb and then ruling out active TB and then may be starting TPT, do you think considering the entire your experience with the Household contact programme of TPT** **as well as the high cost required to do testing. Would there be any potential role of TPT without testing for IGRA as you have already done in household contact for agate workers, what are your perceptions regarding this?**

Based on my 3.5 years of experience which we are currently doing, IGRA testing is not possible for country if we can see like the costing of the test per say. Cy-Tb will be the option and it will be available soon. For testing, government… like PMTPT guidelines also mention that if you are not having IGRA testing available in the district or the state, you can directly go with the chest X-ray. And with the chest X-ray screening, at least you can find out some lesion or X-ray positivity of the individual… based on that if you find some problem in X-ray, you can directly counsel that patient or you can directly bring that patient to the Medical Officer nearby available in the UHC or the government setup and they can check up the symptoms. You are having the X-ray result and also symptoms, both the thing will definitely guide you to take a further decision for the LTBI treatment as well as for the active TB treatment. So, currently we are practicing that if the patient is X-ray positive plus showing symptoms, then Medical Officer can take a call that this patient having symptoms so they can directly collect the sample for directly NAAT and once NAAT comes positive they can initiate on TB treatment. But if X-ray seems normal and patient is also not having any symptoms or signs then they can directly go for the LTBI treatment so we can go with that only till the time Cy-Tb comes.

**What steps do you believe the National TB Elimination Program should take to specifically address the latent TB concerns among agate workers in Khambhat? Are there specific policy changes or resource allocations that could facilitate these steps?**

As I mentioned earlier like this special screening for such group needs to be done that needs to be considered as a priority so does like reducing the spread of such disease. The second thing is the nutrition part of such people who are currently working there, so malnourishment is one of the major cause where the immunity is basically compromised and because of that only some latent infection also converts to the deadliest disease, so, malnourishment needs to be handled rightly. The programme is doing well but their screening and counselling part needs to be strengthened. Currently our field staff is working well, as I mentioned ASHA workers, STS, TBHV and all they are working… they are meeting the patient but they need to be also trained specifically on the counselling part of the agate workers and they should convey all the things in a right manner, well in prior, so that they will be well aware with the such conditions and we can handle them very politely whenever the disease or the prevalence needs to be handle.

**Is there anything else you would like to say that you feel you were not able to say during the interview?**

Thank you very much for the interview actually. We are working with the individuals like who are TB patients in Households but with the working with this special group like you are working with agate workers and all so this is the bigger reason where why the TB incidences are increasing day by day so needs to be handled properly. And, you are in right direction where you can showcase such results where CTD or NTEP can take actions. So, no more suggestions but I think we have covered everything. So all the best for the programme.

**In-depth interview 10: Expert in silicosis and occupational health, 40 years of experience**

**Recording started with permission of the interviewee, after initial discussion on the project and its findings. The interviewee starts expressing his perceptions on the need for starting TB preventive treatment without the need for testing for latent TB infection:**

First of all, what I feel is, we, I mean before we give any recommendations or for that matter even if we, even before we do the study also, we should be very clear in our mind about both the pros and cons. One is we have this public health angle, wherein we see that maximum people should benefit at minimum cost. So that is one angle, in which case we would say that the population is susceptible, so don't just go and give, because then you save time and you have better compliance. That is one way of looking at it.

Another way is now people are becoming very cautious and people are also becoming half-educated. So, and then there are people like our friend from Baroda to educate them. So, I mean they should not, I mean sometimes it can also be seen as, see they are not testing you, you may not have anything but they are still giving you this drug or this, because it is their program and it gives them credit or it gives them numbers or therefore they are using you as guinea pig and if you don't have any TB and you take these medicines, you will have problems. See, these are the side effects. They will highlight, I mean INH and all that, you know there could be some rare thing but they will say this can cause this problem, this can cause that problem and you are being exposed to those side effects and they are not sure.

So, first of all we have to, like you have done whatever study you told me, that what is the prevalence, what is the likely prevalence, that we should have an idea, so that we can also justify it. Second is what are the hazards. Now, there are two ways, one is hazards in the literature, whatever is available to us, but also in the population that we are giving them or we have started it, they should also be monitored for side effects, because going forward that will help us, that this is our drug.

Second is you also see sometimes policy makers also, for different reasons, behave in an irrational way. You must be remembering that for tobacco, I mean the whole world knows what are the effects of tobacco, but in the parliament they had guts to say that whatever hazards of tobacco are being quoted, they are from other countries, there have been no studies in India, you know that. So, because people belong to a particular lobby or whatever, they advocate in this way, so therefore we must be on a sound footing. So, that angle also we have to see and if we say, I personally feel that the side effects would be minimal and the benefits will be more. So, personally I am, I don't want to get bogged down by doing this individual test and all that, because we know, I mean going by the public health this thing, yeah, there would be some small, for example when this COVID vaccine came, who had done this testing and all that, it was an emergency measure. So, now they are coming up this side effect, that side effect and heart attacks and all that, I also don't know how much of that is misinformation and how much of that is technically correct research, we do not know that, but then we took the chance, we took the risk and that has helped us, that has helped us overcome the epidemic, arrest the losses that we were suffering, I mean I very strongly feel that the second wave was very lethal in India, but after that such a lethal thing did not come about and a lot of it could be because of vaccination, so that people would not, so probably we can have the same approach here, because we have a vast population.

As far as numbers of silicosis is concerned, see the employer would never like a silicosis to be reported and so what we saw in Rajasthan, so in 2018 when we were doing the study, we wanted data and all that, the central government, it gave reports in the parliament, very low number of cases, but at the same time when in Rajasthan they started that compensation for silicosis, there are thousands of cases, so far they have compensated almost 15,000 cases, but then you see your records, you see your reports of occupational data and show me 15,000 cases, 5000 cases for that matter, they are not there, even when ILO did that, ILO and DGFASLI, they together did that occupational health profile of India, so in that they also printed the reported cases, so the most common number in that is nil and there also, I mean for 5 years there are no cases of NIHL, then there are 80 cases of NIHL, then again there are no cases of NIHL, how can it come like that, there can be variation, but there are none, suddenly you have a spot and then there are again, that is because these cases were detected, there was something and factory inspector must have said something, the cases were reported, subsequent years again they took care and no cases were reported, so as far as occupational health is concerned, where employer is available, we will never get good data in India, as of now, these things should improve, but that is the present status, so because you had mentioned the number, that is why it came to my mind, and it is a good way.

Second is in India, it is certainly a noble objective to prevent tuberculosis, at the same time we should also somewhere have an objective of preventing silicosis, because you might prevent tuberculosis today by giving this treatment for 2 years, 3 years, 4 years, 5 years, whatever it is, but if his exposure to silica continues, he is going to get silicosis after that. Whether he gets TB or not, his life is limited, and the disease is self-progressive, then even if you stop exposure, his silicosis will continue, so that element also needs to come somewhere. I don't think we have any national program on prevention of pneumoconiosis or silicosis, so somewhere, I mean think of that also, that could also become an offshoot of all this or whatever, somewhere, something, as public health, TB is important, but as NIOH, silicosis is important, and incidentally both go hand in hand, but preventing TB will not make any difference, to their silicosis, so therefore this also has to have some element sooner or later, or advocacy or whatever you can say on silicosis, because otherwise there is no point, I mean you do this, protect them for 2 years from tuberculosis and then they develop silicosis, which is much worse than tuberculosis, and knowing that these people are vulnerable to silicosis, that's why you are giving them TPT, so it's, I mean I know he is developing this, and I am preventing the side thing which is going to come, and allowing the main thing to continue, that is a physician's dilemma, you can say, our occupational health, so that's something, I know I mean, you have a limited project, it will have a limited objectives, so it may not come into that, but as, I don't have those restrictions in my thinking, so I would certainly see that we need to focus on that also.

**Sir, so I mean, apart from overdose detection, do you have any other, you know, suggestions that would, you know, prevent both silicosis as well as tuberculosis.**

Of course, see, as far as prevention of silicosis is concerned, there is only one way, exposure, you stop the exposure, reduce the exposure, eliminate, where elimination is not possible, you create barriers, sees that the intensity of exposure reduces, duration reduces, all those things we have to do, for reducing silicosis, first of all, people have to accept silicosis as a possibility, which people are not willing to do, and that is because of employer, no employer likes to accept any occupational disease, so that is the thing, so of course, we should do awareness, we should do advocacy, we should do sensitization, and I do not know how far Dr. Sarkar has started that test, so his sensitivity, specificity, I don't know, but if you are doing IGRA, then along with that, based on the history, some people could be exposed to that test also, if it is relevant, because in X-ray, it may not be possible, then 300 mA X-ray is not available everywhere, radiologists are not available to interpret it properly, for that matter, what we have seen in silicosis, even this classification and all that, this ILO classification, they repeatedly or periodically conduct, you go and attend one of them, become certified for that, tell Sishodiya, he will tell you, yes, go and attend, I hope you are maintaining coordination with Dr. Sishodiya, he is a very good resource person in that area, and retired like me, so have time, but still engaged because of passion in some or the other activities, but our engagement is not for money, our engagement is for passion.

**What are your perceptions regarding the significantly higher prevalence (58%) of latent TB infection among agate workers compared to the general population (31%)? What explains the near doubling of LTBI in this group?**

Yeah, that is an important finding, that also substantiates the belief that we have, and it is also logical, because see silica, it also reduces your capacity, it also sensitizes you, reduces your immunity, reduces your haemoglobin, reduces your respiratory, affects your respiratory system adversely making you more vulnerable, this was all in theory, but in practice also it is happening, we all know it from theory, but in practice also it is happening, so not only agate workers, but wherever we have such things, where there is a possible exposure, we could consider extending this scheme or this thing, and including them into this process.

**So, we discussed just before we started recording that, yeah, I mean IGRA testing is a bit challenging, you know, it cost, the kids cost a bit, you need a sophisticated laboratory, so any, I mean, of course, I mean, you may not actually know the TPT program, what is happening, but we give upfront TPT to household contacts, without testing for IGRA, but of course we rule out active TB by doing X-ray, as well as based on the symptoms, so any thought on, you know, giving upfront TPT to these agate workers, and not only agate workers, I mean agate workers would be for other project, but there is a large part of silica dust exposed population, so what are your thoughts on, you know, giving upfront TPT, just by ruling out, because given the fact that almost, you know, 2 out of 3 silica dust exposed are testing positive for TB.**

What I feel is, yes, you can give upfront TPT, at the same time, at least a group of population should be subjected to IGRA test, you may not subject all of them, because, see, this is an applied project, this is not only research oriented, you cannot leave it, I will do my research, let the patient die, we cannot have that approach or that attitude, so if you see some benefit, even at half way, you have to forget your project objectives and first take care of the people, because that is the primary ethics anywhere, so you can start this, at the same time, a subsection of the population, whatever is feasible, should continue to undergo this, because that will keep on validating what you are doing, at a later point of time, supposing your IGRA test tells you, in a given population, that no, this is something different, here the prevalence is as good as general population, or may be even less than general population, then you can have a rethink on your this thing, if we don't do that, we will never reach to that level, we should not presume, that is what I feel.

**Right sir, and sir, as I mentioned that the TPT program has silicosis as the vulnerable population, whereas our research says that the prevalence is quite high among the silica dust exposed itself. What is your opinion on silica-dust-exposed individuals as a high-risk group under the NTEP-PMTPT program?**

Yes, yes, certainly, absolutely, there should be no two opinions about that.

**Is there anything else you would like to say that you feel you were not able to say during the interview?**

No, I mean, the principles remain the same, we have to have proper surveillance, earliest diagnosis, and what I feel is, while to a subsection, you must have a rigorous scientific program, to the population at large, you should apply the public health principles, for the benefits, which are obvious, and continue with that, but this subsection, we should run concurrently, as I have already explained my opinion, would validate whatever you are doing, or would correct in the rare case, that it needs correction, so that will give us that part. Policy makers, in general, now what has happened is, somehow, general population does not think TB as a major problem, because for them TB is an old problem, unless they themselves get diagnosed, they don't realize the importance, because it's not in news, it does not have a glamour, I mean, today, if, for example, those 41 workers, who were trapped in that tunnel, you have seen how much was the national attention, you have seen how much resources government has spent, to save those 41 workers, but along with that, there was a news, at the same time, there was a news in the newspaper, that those who are entering these manholes, some 500 or 700 workers have died in last 4 years, that news comes and goes, we are not worried about those 700 deaths, we are worried about these 41, because they are catching your fancy, what about those 700, so same thing is happening with TB, that people get TB, people get treated, people die, I mean, people take it as, in general population takes it as granted, so therefore the policy makers, the opinion makers, and including the opinion leaders like social media, mass media, newspapers, newspaper reporters, we need to sensitize them, so we can't sensitize population as such alone, we have to find out such influencers, and take their might for this.

**In-depth interview 11: State-level official (NTEP), 10 years of experience**

**What are your perceptions regarding the significantly higher prevalence (58%) of latent TB infection among agate workers compared to the general population (31%)? What explains the near doubling of LTBI in this group?**

So, if we look at it, the place where there is silicosis or silica dust is generated, after that, in other cotton industries, our perception is that lung diseases and lung capacity is likely to decrease. If we look at it, in all over the country, the prevalence of TB infection is about 40%. In Gujarat, it is about 37%. So, the place where the prevalence is more, the lifetime risk of TB is 10% in the general population, which is about 40%. In this, it is about 40% to 60%. So, the lifetime risk of TB is more in the place where people are exposed to silica. So, the chances of TB infection are more. And when the incidence of TB is more, if you look at any place, the bacterial load of TB bacilli is more in the atmosphere. And if there is an unhygienic condition, their own food habits or their immunity, malnutrition is also a problem. Because, especially the poor population, they work in the factory. So, their malnutrition status is also weak. So, they have to face a lifetime risk of TB or get infected. And at the same time, there is a lack of cross ventilation there. After that, when they are working, they have to take safety precautions. So, if they don't take safety precautions, silica will go inside. At the same time, TB bacteria will also go inside.

**So, what do you think, the occupation of those people, the work that they do, due to that, has there been a doubling of the incidence of TB infection?**

Yes. So, if there is any kind of dust, whether it is silica dust or any kind of dust, if someone is working in a cotton industry, if someone is working on a construction site, or if someone is working on a temple, or if someone is working on a statue, then there are more chances of getting infected. Because when silica goes inside the lungs, then the capacity of the lungs decreases. And they are most vulnerable to getting infected.

**In your opinion, what factors contribute to the high positivity of latent TB infection among agate workers, especially those engaged in high silica dust work like polishing or chipping (2 times higher) than drilling (65% vs. 52%)?**

Yes. In chipping, the exposure will be more. And at the same time, in chipping the small crystals, they are very small, so there is a maximum chance of getting stuck in the lungs. And it will go inside more during inhalation, so it will get trapped. So, especially when it comes to drilling, it is done single-handedly, a simple hole is made with a machine. So, in that, there is not much dust pollution, but the particles, in chipping, are very small, and if there is more exposure, there are more chances of getting infected. And as there are smaller particles, the small particles go inside more during inhalation.

**Could you elaborate on the challenges faced in conducting LTBI testing among agate workers, considering factors such as the high cost of kits and the need for sophisticated laboratories?**

There are two things, one is, we are doing Mantoux tests, for infection. So, we can do Mantoux tests, but its sensitivity is not as much as IGRA. IGRA, no doubt, it is costly, it is cost-effective, so its cost is more, and it is a little complicated procedure, so it is a little difficult for sample collection also, because we have to take an individual’s whole blood at that time. While Cy-Tb is a new thing, it is similar to the Mantoux test. In that, a simple prick is required, and it can be examined. It’s pilot has been done in Gujarat, and in the near future, its approval will be received through ICMR, so when it comes, it will be more convenient, because any ordinary laboratory technician, or our health worker, can also do it. While in IGRA test, special phlebotomists are required for blood collection, and if it is a periphery area, then it is difficult to do transportation from there. And the sample has to be sent to the place where IGRA is happening, if we send it to such a laboratory, then there are difficulties in managing the transportation charges. So, Cy-Tb is the best option for it, because the pilot we did in Gujarat, its results are good, and it is an easy procedure.

The rest is like this, that we do test and treat strategy and only treat strategy, these are the two things. Now, in general population, it is necessary to test everyone, but according to me, for the vulnerable population, for example, contacts of TB patient, or the place where the silica is generated, according to you, more than 60% to 70% of the workers are positive… so when positivity, the prevalence of infection is more in such vulnerable population, in that, instead of test and treat strategy, we can go for direct treat strategy. But, of course, it is necessary to check, whether they have TB or not, and if TB is ruled out, then TPT should be given directly, according to me.

**What challenges do program functionaries face in providing Tuberculosis Preventive Treatment to agate workers, especially in terms of the availability of Isoniazid and combination drugs like Isoniazid-Rifapentin?**

The challenges in TPT, is the supply of medicines, so it is supplied from the central level, so from the central level, we are given 6H, so the Isoniazid, we have to give for 6 months, and there is an issue in the adherence. We are giving it in contacts of TB patients… generally they say no to take 6H. In private doctors also, there is an issue of acceptance, the private doctor has a fear, that if we give Isoniazid for 6 months, then there will emergence of drug resistance. So we are facing these challenges. And from the national level, the medicines that are supplied, especially in the last one year, there was some constraint. Now in the near future, 3HP will be supplied, which has 12 doses, and these are weekly doses, so chances of acceptance are more. So from the central TB division, if the medicine comes, I think it will be beneficial. So the main challenge is, the contacts of [TB patients in] the private sector, so in that contact, there is less acceptance, because in the child, from 0 to 5 years, when we were giving it to the child, there was no issue of acceptance, as it was a dispersible tablet, it does not affect the child. But in adults when we give TPT in the absence of any symptoms, there is less acceptance. So it is a matter of behaviour change, so the community needs to be aware, along with that, during CMEs, the doctors need to be made aware… even with one-to-one interactions we explain it to the doctors that by giving TPT, there is a reduction in incidence, around 10%, that is what the document says, so it will benefit us.

**According to our PMTPT guidelines, silicosis is considered as a high-risk group. However, diagnosing it is challenging as physicians may not readily suspect it, and there is a lack of training for diagnosing it. In our study, we found a 58% prevalence of infection among the silica dust-exposed population, but when we look at the Khambhat population, where people mainly use the water grip method, dust exposure is significantly lower. Considering that approximately 52 million people are exposed to silica dust nationwide, what is your opinion on silica-dust-exposed individuals as a high-risk group under the NTEP-PMTPT program?**

Yes, it can happen, that when the prevalence is very high, then we just have to rule out TB. After that, without IGRA testing, or without any other tests for infection, TPT should be given directly, as per my opinion. Because, in that, your prevalence itself is high, direct treat strategy can be employed to give TPT and we can reduce the chances of disease conversion.

**In household contacts TPT program, we rule out active TB by X-ray and symptoms. Can the same strategy be used for silica-dust-exposed population?**

Yes, the silica dust exposed group comes under vulnerable population, so, such population can be mapped at the district level, and, that population should be screened at least twice a year. After screening, if there are symptoms, then X-ray, and sputum examination, both should be done together.

**What are the potential solutions to reduce the burden of LTBI among agate workers?**

There are 4-5 things, first, behaviour change communication, so, behaviour change should be done, in the community, so, for behaviour change, all activities can be done. After that, their practices, mask, or wet cloth, if they use mask, silica dust will be inhaled less. And after that, machinery used, in that, in most cases water is poured so as such there are not many issues. But, for the machinery, the engineering department can specially design, or, use something like hood… I have seen some machines, not related to agate industry, but related to other industries… in that, there is no direct dust exposure, like, the drill, if that drill comes with a hood, then less silica dust is generated. It will be generated, but there is no direct exposure. So, in that, hygiene should be taken care of, and other determinants, like TB, other determinants, especially malnutrition, proper hygiene, all these things should be taken care of. They consume alcohol and smoke sometimes, so, they should be addressed, and should be linked to the de-addiction centre, if there is alcohol or tobacco issue. Because, when all this is there, along with that, due to these other determinants, the overall prevalence will be higher. Because it is not that just due to silicosis or silica, they get a 60-70% prevalence… along with it, they might be having these other determinants like diabetes, 20-30% is there, and if diabetic is there, it will be around 30%, so, every year, screening is done for Diabetes, so, Diabetic patients, will have double chances. Silica is already generated, along with that, TB also has more chances, so, twice a year, screening is done and general check-up should be done like hypertension, diabetes… for malnutrition… if there is less than 18 BMI, it is also checked, every year. According to that, appropriate treatment is needed. If BMI is less, it is to be linked to the nutrition centre, or if there is any nutritionist in the government, it is linked to him, and for appropriate balanced diet, or for high protein diet is explained. So, benefits can be obtained. And accordingly smokers are explained for de-addiction. And accordingly hygiene and practices, mouth is not covered… for that they need counselling, and along with that, they will utilize the mask, then the prevalence can be reduced. So, I think, screening is very important, screening should be done for everyone, every year, twice a year, and if X-ray is done in the screening, in the X-ray, if there is the beginning of silicosis can be identified and accordingly, they will be taught exercises, because in many places, when we work in the primary health centre, community health centre, at that time, if there is such a patient, we ask them to change their business, to change his thoughts, so, in the beginning, if there is a problem, future problems due to silicosis and tuberculosis can be stopped.

**Is there anything else you would like to say that you feel you were not able to say during the interview?**

Generally, there are the only things. But the very important thing is that, this population, not only silica exposed, but any lung… because TB is related to the lungs, ok, so, any industry which is related to the lungs, or any industry like cotton industry, or, the place where silica is generated, such as all the casting industries, all the places where the workers work, whether it is an organized sector, or an unorganized sector… in all such places, if screening is done twice a year, of each person, and if there is a symptom, and it seems so, or if the exposure is more than 5 years, then an X-ray is done, then the benefit can be obtained. And, if we talk about artificial intelligence, there is Artificial Intelligence for many diseases. But, if a person, by making a party ready, has an X-ray of silicosis, if he comes to code it in the application of Artificial Intelligence, then his diagnosis becomes easy, because, if you talk about it, if an X-ray comes from a medical officer at periphery level, then it becomes difficult for him to diagnose, because, it is difficult for him to make a difference between miliary TB and silicosis. So, according to me, for the maximum benefit of technology, if something like that can be thought of, then this benefit is for early detection. So, according to what I said, if screening is done, but after screening, if X-ray cannot be read… if there is silicosis in the bilateral lungs, then the diagnosis can be easily done. But, in the initial stages, it gets missed. When the patient comes after 2-5 years, then it increases, when the patient starts having breathlessness and other issues. So, for early detection, if screening is done every year, and using Artificial Intelligence like this, then the benefit is there. So, for that, many number of X-rays are required in the AI portal, coding has to be done… and it has to be installed and uploaded.

**In-depth interview 12: Expert in TB care cascade, 8 years of experience**

**What are your perceptions regarding the significantly higher prevalence (58%) of latent TB infection among agate workers compared to the general population (31%)? What explains the near doubling of LTBI in this group?**

So, I completely agree, when we see that in the general population, the National Prevalence Survey ranges from the higher ranges around 43 to 45 percent, so if you look at the confidence interval of that 38 percent or 31 percentages, it ranges around the maximum percentage around 45 percentages. So, that was in the general population, that was around the randomized population across the country, but when we talk about this worker, these are the workers who do fall somewhere in the high-risk group, in the high risk group of population provided by their socio-demographic conditions. So, it is quite possible that they do have the burden of diseases of mostly communicable diseases just like TB or the TB infection is on the higher side, so it is possible, it is possible.

**Right. So, I mean do you mean to say that or do you want to infer that, it is their work which is probably responsible for the higher latent TB infection prevalence?**

So, over the period what we have observed, there are around 20 to 25 high-risk groups are there in the TB and on the occupational aspects, there are 8 to 9 categories that who are basically as a work, they are categorized for the high risk population, so certainly those occupations who are affected on the clinical aspects of the lung health index, they are more prone for the tuberculosis or possibly the lower respiratory tract infections. So certainly occupation adds additional hazard or additional burden to those population, provided there are other socio-demographic conditions that needs to be looked at and obviously the duration of work, the level of exposure, those kind of things also needs to be considered while we tag them as a high risk population or more prone for the TB.

**In your opinion, what factors contribute to the high positivity of latent TB infection among agate workers, especially those engaged in high silica dust work like polishing or chipping (2 times higher) than drilling (65% vs. 52%)?**

So, first of all let us make the epidemiological triad and that when the agent comes to the host and when we surround the environment, do we have any number of cases there of the TB cases, those who are diagnosed as a TB and that prevalence needs to be established, so some from somewhere they might do have an infection from the TB. The crucial factor is establishing whether there are diagnosed TB cases in the area, indicating TB prevalence. Second thing is the TB possess that when we talk about the exposure of silica dust, so and they do have a more damage to the lungs and that may directly affect with the more prevalence or more chances of having TB infection, whether it is the disease, whether it is the latent TB infection. So obviously the exposure to lung or the damage to the lungs whenever that happens, the person is more prone to have this kind of lung infections.

**Could you elaborate on the challenges faced in conducting LTBI testing among agate workers, considering factors such as the high cost of kits and the need for sophisticated laboratories?**

So, as a project, the project might do have their limitations but for the system, looking at the end-TB strategy and our commitment to the larger aspects of national strategic plan for 2020-2025, we should be going for the advanced stage of diagnostics. And there are challenges, if there are challenges, then system needs to address those challenges through anyway and there is no issue with it for the funds, there are no issue in the availability of diagnostic parts. But there are gaps from the systems side also that needs to be addressed. And those challenges if we go for IGRA or if we go for any other, we need to identify that what kind of interventions we would propose to minimise those challenges or minimise those obstacles. So, in my opinion, we should go for advancement of those diagnostic test and if IGRA is available, we should definitely go for the IGRA and that is the first thing. And if IGRA is not there, we should go for Mantoux or the Cy-Tb, any other options that would be available for diagnosis of TB infection. So that is the first part, but, as per the algorithm, patient needs to be evaluated for TB disease first and X-ray, whether it is X-ray is available or the NAAT testing. So as per the algorithm, NAAT testing and X-rays are mandatory, so both are mandatory as per the algorithm. So first the patient needs to go for NAAT testing, X-ray and then if that comes negative, then the IGRA or any other test, those are available that should be looked for.

**Can you elaborate a bit more on Cy-Tb, if you are aware of how the Cy-Tb program works and what exactly would be involved in testing through Cy-Tb as against IGRA testing?**

So, Cy-Tb is again as a skin sensitivity test and just like the Mantoux test, it is better with the antigen and they do have a limited… so this is more of the availability that we do not have the luxury to have the PPD that was the antigen was used for the Mantoux test. So there is alternative antigen that is under the Cy-Tb, so Mylab has invented or the sorry, they developed those antigens and now approved from the central level, we can go for the Cy-Tb, it is just like a skin sensitivity test. So it does not require a large number of trainings because the staff is already trained for the Mantoux test, so that is it. For the IGRA also, they need to collect the blood only, so it is nothing new when we talk about Cy-Tb or the IGRA.

**What challenges do program functionaries face in providing Tuberculosis Preventive Treatment to agate workers, especially in terms of the availability of Isoniazid and combination drugs like Isoniazid-Rifapentin?**

So, as of now the drugs availability in the whole NTEP part is considered a challenge, so the regular supply chain needs to be ensured, the use of NIKSHAY Aushadhi and tagging with Nikshay is not there within the portal. So, there are lots of systematic system-side challenges, system-side gaps also from the administrator part that leads to the scarcity of drugs availability at taluka level, at the PHC level, at the sub-centre level, at the patient level, so these are the issues. These are the challenges since quite long and that is why these hurdles needs to be overcome, I am not sure that what kind of additional solution we should put in, because the solutions are available, what to do to maintain the availability of drugs, it is widely known, but it is still not happening and that is the challenges from the administrator side, not from the technical side. So, the continuous advocacy, the sensitivity among the political level that needs to be ensured to make drugs available to the patients, these are the issues, because these are the issues on regular TB drugs, first line drugs also and second line drugs also.

**So overall we see that I mean there are some challenges as far as the testing is concerned as well as a lot of challenges as far as the treatment is concerned, so to just to reduce the you know since we are trying to eliminate TB by 2025, you know just to reduce the number of burden for tuberculosis in India, what is your opinion on getting upfront TPT, you know just by ruling out through x-ray and 4S symptoms to silica dust exposed population?**

See, so looking at the prevalence rate and notification rate across the India, we could achieve a higher number since the COVID, that is the first thing about the notification but when we look at the data and when we look at the trends of incidence rate of TB among general population, I am not talking about infection but the disease part, we are still missing the cases. So first thing is that we are still not having the entire mapping of all cases that needs to be diagnosed and when we talk about cases, those cases are going for the more infection among their contacts, among the general population. So that is the first thing that we need to ensure and how well we can identify those missed cases and get them into the Nikshay, into the system, that is the first thing. Second thing is if we want to reduce a TB infection, we need to create an algorithm, we need to put the technology in place, so there are AI diagnostic algorithms are there, softwares are there, that can easily diagnose through the X-ray that patient is having TB or not and then ruling out those with the support of NAAT, we can create more structured algorithm for the eligible patient for TPT, that is the second thing. Third thing to start the upfront TPT, I am not in favour much, because the patient needs to first diagnose for the TB, that is the first thing. Because if we go for the upfront TPT that will create additional burden of drug resistant TB in future. Because as you see the recent trend of Bedaquiline resistance is now increasing, recent Lancet study, the South African study says that there are increased number of Bedaquiline resistance are there, so we do not have that kind of luxury to go for any upfront treatment for any high risk population, we should create upfront diagnostic mechanism without going for other options, so if the person is going for NAAT testing, if the person is going for X-ray simultaneously, why cannot we take the IGRA also, so if they do not have to go on the second time that yes, the patient is negative, let us go for the IGRA, that should not be there. All the diagnostic test needs to be go simultaneously and essential diagnostic list needs to be expanded for the PHC and for the CHC and that can include the IGRA test. Right now, it is at the district-hospital level, so the CHC-level or the PHC-level, health and wellness centre should have diagnostic test, essential diagnostic test list as a IGRA within their list. So the upfront TPT that is the, that level needs to be used cautiously because the upfront treatment for any kind of antibiotics is not recommended, so that is my opinion.

**In household contacts TPT program, we rule out active TB by X-ray and symptoms. Can the same strategy be used for silica-dust-exposed population?**

So, yes we can replicate those algorithms within this population and it is easy because the algorithm obviously, first tells about to ruling of the active TB and then start the upfront TPT, if the TB infection is there, so same algorithm needs to be goes for any high risk population, not only the silica dust or not any agate workers, any brickiln workers or those kind of all occupational risk factor, the same algorithm needs to be imparted.

**As per your expertise and experience, what potential solutions can be implemented to reduce the burden of TB infection among agate workers?**

So, these are the holistic approach needs to be looked at, not per se for the TB part, they do have other problems also, the skin disease, the communicable disease, there are other malnutrition, those kind of holistic approach needs to be established. They might be having the communicable disease or the non-communicable disease also. So, patient-centric approach rather than for the disease-centric approach, we should go for the patient-centric approach and treat any high risk of population, whether it is a silica dust exposure or Bricklin workers or the layman worker or the resident from slums or those who do not follow the hygiene practice well. So those kind of population needs to be considered with the holistic approach and patient-centric approach needs to be imparted. So, while diagnosing the TB, we should not ignore other comorbidities or other options of diagnostic facility or they should be given weightage for all the things, that is the first thing. Second thing is mapping at the NTEP level, there are certain pockets that are high risk for occupation, they do have prison mapping also but for the unorganized sector in the states, they conduct pulse polio, they conduct the measles catch-up campaign, they conduct the VHND day. So, for the TB part, they can use those other program opportunities and then can expand their mapping for these occupational unorganized sectors because in the pulse polio or in the any VHND day sessions, all populations are being covered by the government. So, why not we should go for this mapping of this population, that is the second thing. Third thing again, the patient-centric approach not for the program-specific approach or the disease-specific approach needs to be imparted. The patient-centric approach needs to be expanded with the help of newer health and wellness centres where CHO can be guided to follow SOPs for all the diseases, not only the TB, not only for leprosy, not only for high risk pregnancy. Let us go for SOPs of all clinical conditions starting from community health officers or front line workers to medical officers, that is the second thing. And fourth thing, we had enough focus on comprehensive primary health care, but we forgot the secondary-level of health care services. When we talk about the secondary-level of health care services, we are missing the specialist cadres at the CHC level. We had pushed a lot for the health and wellness centre establishment at the higher level. But what if it goes beyond the clinical skills of a medical officer, we require specialists at the CHC level and till the date we only have 20 percent or 30 percent availability of specialists at the CHC level or the SDH level. So, when we talk about the escalation matrix for the referral part, we need to strengthen the secondary care health facilities also and that is the thing. And third thing for this agate worker specifically I want to say that when we talk about the silica workers or the occupational high risk population, let us find first the patient who does have a TB disease and patient do have a TB infection. Let us create a registers or let us create an active case finding momentum for these populations because in the year, twice a time we used to conduct ACF. So, do we need to have additional efforts in those mapped areas? That is the question. So, in the ACF can we create additional sections to include these mapped areas and that needs to be updated every year just like we do for the pulse polio, plus like we do for the MR campaign, those machine algorithms, those are the aspects and that way we can create an additional momentum to these areas.

**What is your opinion on silica-dust-exposed individuals as a high-risk group under the NTEP-PMTPT program instead of silicosis?**

Of course, the NTEP guidelines the NSP already suggested to go for the patient-centric approach whether it is silica dust, whether it is the Bricklin workers, whether it is any kind of occupation part or the comorbid population. So, it is not about a particular group that needs to be focused. The NSP is clear, the NTEP is clear that any person, whether it is any kind of socio-demographic profile, needs to go through this TB diagnostic algorithm. So, they are clear they do have that kind of algorithm that can cover all the population, all the people irrespective of any occupation. So, if you want to suggest to go for silica dust or if you go for the any kind of adding more number of high-risk group in my opinion if we are going to apply a patient-centric mechanism it does not matter, it does not matter. So, the silicosis or the silica dust exposed. I am not sure the medical officer does have that kind of depth of knowledge also or the administrator does have that kind of tendency so they may consider all the patients then they should, they should. That is my opinion.

**What steps do you believe the National TB Elimination Program should take to specifically address the latent TB concerns among agate workers in Khambhat? Are there specific policy changes or resource allocations that could facilitate these steps?**

So, till the time what we did we mapped the we increased the number of quantifications through the public health sector in the initial years. After 2015 when we go for the PPSA policy we try to capture those patients who are in the private sector per se but still we are not sure that how many cases at each care casket are being missed out or the dropouts. So, the first thing that we need to ensure or the NTEP needs to focus that at the pre concentration phase or at the diagnostic phase or at the treatment phase how many patients are being dropped off. So, those dropped off patients are creating more infections or the disease. So, that is the first thing that we need to ensure or we need to create a strategy or we need to create a monitoring mechanism where we can capture these numbers. That is the first thing. Second thing is there are many issues within the system side that the diagnostic upfront aspects, the treatment, the availability of drugs those core issues are still pertinent since couple of years and if we are not going to address those issues we might be having still active cases even after the treatment that is the second thing. And third thing if you look at the India TB reports, if you take the global TB reports we are having the highest number of relapse cases, the recurrent TB patients. We do not have any kind of capacity to establish how many times this person is being reported as a TB patient or how many times the person has relapsed. So, Nikshay does have that kind of facility to report only, but not to calculate the number of exposures or number of episodes of TB. So, that kind of information would give us more reasons to have an exposed population among the general population. And last thing at the end of the treatment in the PTB or the EPTB patients or the DRTB patients the culture confirmation is still missing at the end of the treatment. Most of the time patients do not have sputum or patients do not go for the clinical examination. So, whenever the patient completes the medication the program reports them as a treatment completed and those definitions need to be revisited when we go for the successful outcome. We only go for treatment. So, we should go for the cured TB patient not only for possibly completing the medication, but also the clinical evaluation at the end of the treatment should be there.

**Is there anything else you would like to say that you feel you were not able to say during the interview?**

So, my only part is also that if we are, if the information is there that can diagnose the latent TB infection we should also go for the TB patients who are having the number of episodes of TB. That is the first thing that we can also identify and can we create some kind of research or the genomic mapping through that we can identify the index case at the advanced level. If we can diagnose that kind of mechanism, who would be the index case among certain settlements that got the first TB infection or the disease? So, that way we can create surveillance mechanisms on the certain population. That is the first thing I would like to say. And second thing is we do have a SRS system and we do have an NFHS system. Can we create surveillance sites for TB? We are on the verge of eliminating TB just like filariasis. We create surveillance sites to collect the blood samples to monitor the certain number of patients. Can we create surveillance sites for TB? And through that we can generate more intellectual data for the particular population. And third thing, we should also invest in a patient adherence weight because when we go for 3HP or when we go for 6H the patient is not very comfortable for treatment adherence for say, they do complain about ADRs and they do complain about many of the other like physical complains that too are not related to drug reaction also. So, what is the best way you can create a treatment adherence without a much heavy ADRs. So, can we create that kind of intervention for populations and take those learnings for the programmatic part that how best we have managed the 100 TB patient at the end of the day that these 100 patients without any complications have completed the treatment. And what are those learning we have applied to the course of treatment? The longitudinal studies for the drug adherence that can be additional aspects on the public health interventions. That’s all.

**In-depth interview 13: Taluka lead TPT Program (Khambhat), 1.5 years of experience**

**What are your perceptions regarding the significantly higher prevalence (58%) of latent TB infection among agate workers compared to the general population (31%)? What explains the near doubling of LTBI in this group?**

There might be one reason within this doubling, that is, firstly, the object that is being used more here is Agate. Some individuals, especially those who don't go to Agate factories, still use these objects at home. So, when a person goes to the factory alone, and when the person brings it home, another 4 individuals are in contact. Since the dust particles are small around, and cleanliness of the house, all these things together can lead to an increased possibility. If you get positive, this might be the only reason.

**In your opinion, what factors contribute to the high positivity of latent TB infection among agate workers, especially those engaged in high silica dust work like polishing or chipping (2 times higher) than drilling (65% vs. 52%)?**

The thing about making a tree [ornamental] or a vessel [ornamental], it can happen both within a closed room or sitting outside the house. So, even in this case, the possibility increases. When dust particles fly around more, they tend to enter the body more. So, the possibility increases.

**Could you elaborate on the challenges faced in conducting LTBI testing among agate workers, considering factors such as the high cost of kits and the need for sophisticated laboratories?**

Now when we talk about this IGRA, the first thing is that it is a costly test, and convincing the public here is a tough job. The Chunara community here doesn't find meaning in all these things. When something happens, then we'll see. We should try to minimize this. So, I believe that instead of IGRA, we should do an X-ray for TPT and rule out by symptom screening, then start the medication. If we do it this way, it won't be too expensive, and people will be convinced sooner because in IGRA, workers also need to give blood samples, and a little way to see the results of IGRA. So, it will be better to rule out X-ray and screening and give medication, this will be more convenient.

**What challenges do program functionaries face in providing Tuberculosis Preventive Treatment to agate workers, especially in terms of the availability of Isoniazid and combination drugs like Isoniazid-Rifapentin?**

As for the availability of TPT, it's true that we have already convinced many people to undergo screening and X-ray, keeping them ready for treatment. However, if we fail to provide medication on time, these people will lose confidence, and it may seem to them that since the index patient has been cured, there's no need for them to take medication. Therefore, it is crucial to ensure the availability of TPT. If the government provides us with the medication, we can start TPT. But if we don't have stock, we need to figure out how we can provide the medication.

**You have been involved in the TPT program for household contacts for quite some time. Are there lessons from the TPT program for household contacts (currently ongoing program being managed by Alert India NGO) that can be applied to agate workers?**

One thing we've learned is that antibiotics can have beneficial effects on the public. We can apply this knowledge to convince Agate workers as well. TPT can benefit household contacts, such as individuals who are underweight, and those who take the 6H regimen can gain weight, which can lead to a better response from household members. Thus, we can enhance the program by emphasizing its benefits.

**We give two regimens - 3HP and 6H, what difference have you observed in your experience between the two, and how can we better explain to patients to increase acceptance?**

When we compare 6H and 3HP, 6H is taken for 6 months, while 3HP is taken for 3 months. In my experience, I found 3HP to be more effective because it is a weekly dose, and patients can be convinced more quickly to take it regularly. Since it's only 12 doses, patients find it easier to comply. On the other hand, with 6H, patients might feel a bit uncomfortable taking it regularly for a longer duration. So, 3HP seems to be more effective because patients are convinced more quickly, and it's easier for them to take regularly. Although there may be some adverse drug reactions (ADRs) with 3HP, it's still better accepted by people. Therefore, 3HP appears to be more effective.

**Could you elaborate more on ADRs and what can be done to prevent them?**

ADR stands for Adverse Drug Reactions. In this, when a patient is given medication 3HP, three months regimen to take, what happens is that vomiting can occur with 3HP, the person may feel anxious, there may be a slight to moderate reddish color in the urine. These are related to the medication but are normal. Other than these symptoms, there is nothing else significant in ADRs. In ADRs, if there is talk of vomiting, then if the medication is taken and immediately vomiting occurs, then the patient will inform us immediately. Then we can say that since you are taking three pills together, instead, take one pill, then after 5-10 minutes take the second pill, then after 5-10 minutes take the third pill, so that the dose does not go into the body at once, then the chances of these ADRs decrease. Another thing is that if this medicine is taken after dinner, then it is more likely to occur in the middle of the night, then if taken after dinner, it can cause more trouble. As if taken in the morning, then it may work during the day, so there may be more trouble in the morning. For example, keep a little gap in between, 10-15 minutes, so that these people may have fewer ADRs thereafter.

**What are the potential solutions to reduce the burden of LTBI among agate workers?**

In terms of change, we can't say anything about their business, but what we are doing for TPT for TB, we should give antibiotic medicine to them, and convince these people to take the medicine. If these people take more damage, then it will decrease, and if it decreases, then it will not increase further, we can say that we can prevent it. And we can't say anything about their business because this is the same job for these people, so these people will do what they don't leave. So we have to become active and work.

**What steps do you believe the National TB Elimination Program should take to specifically address the latent TB concerns among agate workers in Khambhat? Are there specific policy changes or resource allocations that could facilitate these steps?**

Now, if we go into improvements, then within it, the transition model is going on, and if the government staff is also involved more in it, then more work will be done, these people will soon agree and take regular medicine, and it [burden of latent TB infection] will be less likely to increase. Because when we give TPT, then latent TB will gradually increase, and then it will be less, because we give antibiotics to these people, so there will be no further increase, and an incremental decrease will be observed.

**Is there anything else you would like to say that you feel you were not able to say during the interview?**

The reason to reduce Latent TB Infection can be one, if government staff gets more involved, then everyone should be involved, because some people are such that they only believe in orders, others do not believe. Such as if there is an MPHW, then more attention should be paid to CHO, then more attention should be paid to MPHW. ASHA worker will come with MPHW during visits, and if more attention is paid, then more involvement can benefit everyone. Because these people work only when everyone agrees, if government staff is not involved, then this thing is not possible. We can't pinpoint anyone because it is not the work of one person, if everyone comes together and does it, then this thing will be resolved quickly, if one person runs, then this thing will not be resolved.
