# Supplementary material for: Mixed methods study on latent tuberculosis among agate stone workers and advocacy for testing silica dust exposed individuals in India
Source: Sci Rep. 2024 Jun 15;14:13830. doi: 10.1038/s41598-024-64837-4 (PMC11180111; doi:10.1038/s41598-024-64837-4)
Supplement: Supplementary file 5 — Supplementary Information 5. [file 41598_2024_64837_MOESM5_ESM.docx]

**Calculation of the Standard of Living (SLI) index:**

The SLI index was calculated based on the ownership of the assets by the households. We used the following scoring for the SLI index in our study.

1. *Type of house and number of rooms:* a score of 4 for a house which has walls and roof made of cement concrete and has ≥2 rooms; a score of 3 for a house which has walls made of cement concrete but the roof is made of material other than cement concrete and having ≥2 rooms; a score of 2 for a house which has walls made of cement concrete but the roof is made of material other than cement concrete and having 1 room; a score of 1 for a house which has walls as well as the roof made of material other than cement concrete, irrespective of the number of rooms [a pucca house with ≥2 rooms gets a score of 4, a pucca house with <2 rooms get a score of 3, a semi-pucca house with ≥2 room gets a score of 3, a semi-pucca house with 1 room gets a score of 2, a kutcha house gets a score of 1].
2. *Own house:* score of 4 for owning the house, otherwise 0.
3. *Separate kitchen:* a score of 3 for a house with a separate kitchen, otherwise 0.
4. *Liquified Petroleum Gas (LPG) or natural gas as cooking fuel:* score of 1 for a household using, otherwise 0.
5. *Land usable for agriculture:* score of 4 for owning such a land, otherwise 0.
6. *Own livestock, herd or farm animals:* score of 3 for owning, otherwise 0.
7. *Television, bicycle, mobile:* score of 1 each for owning them, otherwise 0.
8. *Refrigerator, motorcycle/scooter, air conditioner/air cooler, washing machine:* score of 2 each for owning them, otherwise 0.
9. *Car/truck/tractor:* score of 4 for owning one, otherwise 0.
