# Supplementary material for: Mixed methods study on latent tuberculosis among agate stone workers and advocacy for testing silica dust exposed individuals in India
Source: Sci Rep. 2024 Jun 15;14:13830. doi: 10.1038/s41598-024-64837-4 (PMC11180111; doi:10.1038/s41598-024-64837-4)
Supplement: Supplementary file 6 — Supplementary Information 6. [file 41598_2024_64837_MOESM6_ESM.docx]

Supplementary Table S1: Contributing factors for high burden of latent TB infection among agate-stone workers in Khambhat

| **Categories** | **Codes** | **Description** |
| --- | --- | --- |
| Work environment factors | Silica dust exposure | Exposure to silica dust in the workplace, a prevalent factor in the agate industry, contributing to increased risk of latent TB infection among workers. |
|  | High workplace density | The overcrowded work environment that enhances the probability of TB transmission among agate-stone workers. |
|  | Low ventilation | Inadequate ventilation in workplaces, fostering the accumulation of airborne contaminants and potential TB transmission. |
|  | Inadequate safety measures | Lack of proper safety protocols and measures, potentially exposing workers to conditions conducive to latent TB infection. |
|  | Alternate employment scarcity | Limited availability of alternative employment opportunities, compelling workers to persist in high-risk environments. |
|  | Employer exploitation | Instances of employer exploitation, potentially leading to unfavorable working conditions and increased vulnerability to TB. |
| Socioeconomic factors | Low socioeconomic status | Economic factors contributing to a lower socioeconomic status, which may be linked to an increased risk of TB among agate-stone workers. |
|  | Poor nutrition | Suboptimal nutritional conditions, which may compromise immune function and contribute to latent TB infection. |
|  | Low immunity | Weakened immune systems due to various factors, potentially heightening susceptibility to TB infection. |
|  | Overcrowding | Living and working in crowded spaces, increasing the likelihood of TB transmission among workers. |
|  | TB stigma | Stigmatization related to TB, potentially impacting workers' willingness to seek healthcare and adhere to preventive measures. |
|  | Addictive habits | Habits such as tobacco and alcohol use that may compromise overall health and immune function, potentially influencing TB susceptibility. |
|  | High baseline TB prevalence | Existence of a high baseline TB prevalence in the community, contributing to the overall burden of latent TB infection. |
| Work-type dust variations | High dust in polishing | Increased dust generation during polishing tasks, contributing to heightened exposure and potential latent TB risk. |
|  | High dust in chipping | Elevated dust levels during chipping activities, potentially increasing the risk of latent TB infection. |
|  | Fine dust in polishing | Presence of fine dust particles during polishing processes, adding to the respiratory risk faced by agate-stone workers. |
|  | Fine dust in chipping | Generation of fine dust during chipping tasks, contributing to the respiratory challenges experienced by workers. |
|  | Polishing uses large stones | The use of large stones during polishing, generating more dust in the workplace. |
|  | Drilling uses small stones | Utilization of small stones during drilling tasks, generating lesser dust in the work environment. |
|  | Low dust in drilling | Reduced dust generation during drilling activities compared to polishing and chipping. |
|  | Water used in drilling | Incorporation of water in drilling processes generating reduced dust. |
| TB disease challenges | TB disease vs. infection challenge | Challenges in distinguishing between active TB disease and latent TB infection among agate-stone workers lead to confusion while counselling them for accepting latent TB infection testing and adherence to TB preventive treatment. |
|  | Reluctance for health checkup | Workers' hesitancy or resistance toward undergoing health checkups, hindering early detection of TB. |
|  | Low awareness | Limited awareness among workers regarding TB, potentially contributing to delayed healthcare seeking and preventive measures. |

Supplementary Table S2: Mitigation strategies for high burden of latent TB infection among agate-stone workers in Khambhat

| **Categories** | **Codes** | **Description** |
| --- | --- | --- |
| Dust control measures | Water use in work | Incorporation of water in work processes to control dust, reducing the risk of respiratory exposure among agate-stone workers. |
|  | Exhaust systems | Implementation of exhaust systems to effectively capture and remove airborne dust particles from the work environment. |
|  | Silicosis prevention | Measures and interventions aimed at preventing the development of silicosis. |
|  | Engineering control | Implementation of engineering controls such as having specially designed hoods to reduce or eliminate exposure to silica dust, enhancing workplace safety. |
|  | PPE | Utilization of personal protective equipment (PPE) to safeguard workers from respiratory exposure to silica dust. |
| Workplace health and safety measures | Periodic medical examination | Regular medical examinations to monitor the health status of agate-stone workers and identify potential TB cases early on. |
|  | Separate workspaces | Allocation of distinct workspaces to minimize cross-contamination and reduce the risk of TB transmission among workers. |
|  | Employment opportunities | Creation of alternative employment opportunities to reduce dependency on high-risk agate-stone work. |
|  | Worker registration | Systematic registration of workers to facilitate tracking, monitoring, and ensuring access to healthcare services. |
|  | Employer responsibility for worker health | Acknowledgment of employer responsibility for the health and safety of agate-stone workers, promoting a supportive workplace environment. |
|  | Strict factory regulations | Implementation and enforcement of stringent regulations within agate factories, including cottage industries, to ensure compliance with health and safety standards. |
|  | Infection testing by owners | Initiative by factory owners to conduct regular infection testing among workers, contributing to early detection and prevention of TB. |
| Awareness and counselling strategies | Community awareness | Initiatives to raise awareness within the community about TB, its risks, and preventive measures, fostering a culture of health consciousness. |
|  | Media for mass awareness | Utilization of various media platforms such as television, radio, and YouTube, for mass communication to disseminate information about TB prevention and treatment. |
|  | Celebrity endorsements | Involvement of celebrities to endorse and promote awareness campaigns related to TB among agate-stone workers. |
|  | Influencers for awareness | Engagement of local influencers such as *‘bhuva’* (traditional faith healer), or *‘baba’* (local term for saints) to effectively convey TB-related messages and encourage preventive actions. |
|  | Counselling for TPT acceptance | Counselling sessions focused on promoting the acceptance and adherence to TB preventive treatment (TPT) among agate-stone workers. |
|  | Counselling for testing | Guidance and support through counselling to encourage agate-stone workers to undergo latent TB infection testing. |
|  | Health worker involvement in counselling | Involvement of healthcare workers in counselling sessions to provide guidance on latent TB testing and TB preventive treatment (TPT). |
|  | Diet counselling | Provision of nutritional counselling to agate-stone workers, enhancing overall health and immune function. |
|  | Increase private practitioners' awareness | Initiatives aimed at increasing awareness among private practitioners to address concerns regarding potential emergence of drug resistance and to underscore the role of TB preventive treatment in reducing the incidence of active TB cases. |
|  | Community participation | Encouragement of active participation from the community in TB awareness initiatives. |
| Enhanced case detection and surveillance | Timely treatment post-diagnosis | Ensuring prompt initiation of treatment following the diagnosis of latent TB infection, minimizing the risk of active TB disease and subsequent transmission. |
|  | Capture missed private cases | Strategies to identify and address cases of TB that may have been missed during private healthcare consultations. |
|  | Identify missed cases | Systematic approaches to identify cases of TB that may have been overlooked or not reported in previous surveillance efforts. |
|  | Agate worker surveillance | Implementation of targeted surveillance specifically tailored for agate-stone workers to monitor and manage TB cases effectively. |
|  | Active case finding | Proactive efforts to identify and diagnose TB cases actively within the agate-stone worker community, improving early detection. |
|  | Symptom screening | Regular screening for TB symptoms among agate-stone workers to facilitate early identification and intervention. |
|  | BMI screening | Body mass index (BMI) screening to assess nutritional status and identify potential indicators of TB among agate-stone workers. |
|  | X-ray screening | Utilization of X-ray screening for early detection of silicosis among agate-stone workers and thereby preventing future development of TB. |
|  | TB surveillance sites | Establishing dedicated sites for systematic TB surveillance aligned with the sample registration system (SRS) and national family health survey (NFHS), facilitating targeted monitoring and management of cases within distinct population groups. |
|  | Mapping via existing programs | Optimizing resource utilization and enhancing coverage by integrating TB mapping activities into established healthcare initiatives like national polio immunization rounds, measles catch-up campaigns, and village health-nutrition day (VHND) events. |
|  | Index case genomic mapping | Utilization of genomic mapping techniques to trace and study the index cases of TB. |
| Improve healthcare infrastructure | Comprehensive patient focus | A holistic patient-centric approach that prioritizes the overall well-being of patients, ensuring comprehensive healthcare coverage and support. |
|  | Enhance secondary healthcare | Strengthening secondary-level healthcare facilities to improve the quality and accessibility of healthcare services. |
|  | Mitigate systemic challenges | Addressing systemic challenges such as drug availability and having a Nikshay online portal module for recurrent TB cases so as to record the number of episodes of TB. |
|  | Dedicated health centre | Establishment of a dedicated health centre specifically catering to the healthcare needs of agate-stone workers. |
|  | Equipped TB clinics | Ensuring that TB clinics are well-equipped with nucleic acid amplification testing (NAAT), latent TB testing using interferon gamma release assay (IGRA), and X-ray facilities to provide optimal care. |
| Improve government engagement | Senior officials' visits | Involvement of senior government officials in on-site visits to understand the challenges and contribute to effective solutions. |
|  | Political support | Securing political support and commitment to addressing the health challenges faced by agate-stone workers, including TB prevention. |
|  | Government support for workers | Ensuring comprehensive government support for the health and well-being of agate-stone workers, particularly in the context of TB prevention. |
|  | Multi-sector coordination | Coordination and collaboration across multiple sectors to address TB-related challenges comprehensively. |
|  | Balanced task assignments | Implementing a balanced allocation of tasks and duties among TB health workers, including field visits, TB treatment card management, data entry into the Nikshay online portal, and other essential responsibilities. |
|  | Stability in program changes | Promoting stability and consistency in programmatic changes to facilitate better planning and implementation of TB prevention strategies. |
|  | De-addiction centre linking | Establishing links with de-addiction centres to address alcohol use issues among agate-stone workers, contributing to overall health improvement. |

Supplementary Table S3: Barriers in testing for latent TB infection and TB preventive treatment (TPT) implementation among agate-stone workers in Khambhat

| **Categories** | **Codes** | **Description** |
| --- | --- | --- |
| IGRA test challenges | Costly IGRA test | High expenses associated with the Interferon-Gamma Release Assay (IGRA) test, posing a financial barrier for agate-stone workers. |
|  | Limited IGRA availability | Restricted accessibility to the IGRA test, limiting the opportunities for agate-stone workers to undergo this specific diagnostic procedure. |
|  | Blood collection challenge | Difficulties and challenges related to the collection of blood samples required for the IGRA test, affecting the testing process. |
|  | Cold chain maintenance | The need for maintaining a consistent cold chain during the transportation and storage of IGRA test samples, adding logistical complexities. |
|  | Complex lab procedures | Complicated laboratory procedures, such as ELISA, associated with the IGRA test, potentially leading to delays or difficulties in obtaining test results. |
|  | Transportation charges | Additional costs related to the transportation of samples for the IGRA test, contributing to the overall financial burden on agate-stone workers. |
|  | Testing reluctance among healthy | A general hesitancy among seemingly healthy agate-stone workers to undergo latent TB infection testing, including the IGRA test. |
| TPT regimen issues | Long 6H regimen | Acknowledging the daily Isoniazid (H) for 6 months as a potentially longer regimen for TB preventive treatment (TPT) as compared to the alternatives available. |
|  | LFU: 6H regimen | Due to the longer 6 months regimen, 6H has loss to follow-up (LFU) as a potential challenge. |
|  | Short 3HP regimen | Promoting the use of a shorter weekly Isoniazid-Rifapentin (HP) for 3 months regimen for TPT, potentially improving adherence and completion rates among agate-stone workers. |
|  | ADR: 3HP regimen | Acknowledging and managing potential adverse drug reactions (ADR) associated with the 3HP regimen, ensuring safe and effective preventive treatment. |
| TPT implementation challenges | TPT supply challenge | Obstacles related to the availability and consistent supply of TB preventive treatment (TPT), affecting its accessibility for agate-stone workers. |
|  | Insufficient TPT manufacturers | Limited production capacity or insufficient number of manufacturers for TPT drugs, leading to challenges in meeting the demand for TB preventive treatment. |
|  | Underestimated TPT requirement | Instances where the demand for TPT is underestimated, resulting in inadequate planning and provision of preventive treatment for agate-stone workers. |
|  | TPT reluctance among healthy | Aversion or hesitancy among apparently healthy agate-stone workers to accept TB preventive treatment, possibly due to concerns about consuming medicines in the absence of any signs or symptoms. |
|  | Drug resistance fear | Apprehension and fear among private practitioners regarding the potential development of drug resistance associated with TB preventive treatment. |
|  | Adverse drug reactions | Concerns and experiences related to adverse drug reactions associated with TB preventive treatment, influencing the willingness of agate-stone workers to adhere to preventive treatment. |

Supplementary Table S4: Proposed measures for improving testing for latent TB infection and TB preventive treatment (TPT) implementation among agate-stone workers in Khambhat

| **Categories** | **Codes** | **Description** |
| --- | --- | --- |
| IGRA testing infrastructure | Government aid for testing | Advocating for governmental support and financial assistance to enhance the availability and accessibility of Interferon-Gamma Release Assay (IGRA) testing for agate-stone workers. |
|  | Widespread IGRA access | Promoting initiatives to broaden the reach of IGRA testing, ensuring that a larger proportion of agate-stone workers can easily access this diagnostic procedure. |
|  | Tendering for IGRA kits | Implementing a procurement strategy through tendering to efficiently acquire IGRA kits, optimizing the supply chain for testing resources. |
| Skin sensitivity testing options | Cy-Tb test alternative | Exploring alternative skin sensitivity tests like the Cy-Tb test as a viable option for diagnosing latent TB infection among agate-stone workers. |
|  | Easy-to-perform Cy-Tb | Emphasizing the simplicity and ease of performance associated with the Cy-Tb test, making it a practical choice for widespread application. |
|  | Mantoux test alternative | Considering alternative options such as the Mantoux test for skin sensitivity testing, providing diverse choices for effective diagnosis. |
|  | Mantoux: low sensitivity | Acknowledging the potential limitations in sensitivity associated with the Mantoux test. |
| Focused testing strategies | Targeted testing for agate workers | Advocating for a targeted approach to latent TB infection testing, specifically tailored for agate-stone workers. |
|  | Evidence-based high-risk group inclusion | Generating more evidence on silica-dust-exposed individuals for defining it as a high-risk group for latent TB infection testing. |
|  | Testing for dust-exposed | Directing latent TB infection testing initiatives towards individuals consistently exposed to dust, recognizing the occupational risk factors associated with latent TB infection. |
|  | Silica-exposed as high-risk group | Recognizing silica-exposed individuals as a priority population vulnerable to latent TB infection, shifting focus from silicosis to occupational silica exposure in guiding testing strategies for latent TB infection. |
| Upfront TPT decision | Upfront TPT for dust-exposed | Advocating for an upfront decision to provide TB preventive treatment (TPT) to individuals exposed to silica dust in occupational settings where the prevalence of latent TB infection is very high, foregoing the conventional test-and-treat approach with IGRA testing. This shift aims to expedite intervention and mitigate the risk of TB transmission and progression among exposed workers, moving towards a direct treatment strategy. |
|  | Low risk, high benefit | Emphasizing the favorable risk-benefit ratio associated with upfront TPT decisions, underscoring the potential positive impact on agate-stone workers' health. |
|  | Upfront TPT caution | Encouraging a cautious approach in upfront TPT decisions, considering the risk of emergence of drug resistance. |
|  | Rule out active TB | Prioritizing the evaluation and exclusion of active TB before initiating TPT, ensuring the appropriateness of preventive measures. |
| Optimize TPT administration | Information on common ADRs | Providing comprehensive information on common adverse drug reactions (ADRs) associated with TPT, enabling informed decision-making and adherence monitoring. |
|  | Strategic timing for TPT | Strategic timing for Tuberculosis Preventive Treatment (TPT) administration is crucial for mitigating adverse drug reactions and enhancing treatment adherence among agate-stone workers. This strategy involves considering various factors to optimize the TPT regimen. For instance, administering TPT after dinner may help alleviate adverse drug reactions during sleep, thereby improving tolerability and compliance. Additionally, implementing a dosing schedule that includes a 5-15 minute gap between each of the three tablets of the 3HP regimen can reduce the likelihood of vomiting, enhancing treatment acceptability. Furthermore, scheduling TPT administration on the day of the weekly off (holiday) for agate-stone workers ensures minimal disruption to their work days, facilitating better adherence to the treatment regimen. |
|  | Supervised treatment for compliance | Incorporating supervised treatment sessions to improve adherence and compliance with the prescribed TPT regimen among agate-stone workers. |
|  | First-month TPT follow-up | Particularly for those undergoing the 3HP regimen, ensuring consistent adherence during the first month, spanning the initial four doses, greatly enhances treatment completion rates. Vigilant follow-up and support during this crucial phase mitigate risks of adverse drug reactions (ADRs) and loss to follow-up, establishing a routine and fostering a sense of progress towards treatment completion. Thus, prioritizing thorough counselling and sustained follow-up during the initial 4-5 weeks significantly improves treatment outcomes. |
|  | ADR mitigation best practices | Implementing best practices for mitigating and managing adverse drug reactions (ADRs) associated with TPT, enhancing overall treatment safety. Additionally, longitudinal studies on drug adherence can supplement public health interventions, providing further depth to our understanding and improving overall treatment outcomes. |
| Streamline TPT procurement | Advance procurement | Engaging in advance procurement strategies to secure a consistent and timely supply of TB preventive treatment (TPT), minimizing potential disruptions. |
|  | Continuous TPT stock | Ensuring continuous and sufficient stock of TPT drugs, preventing stockouts and facilitating uninterrupted provision to agate-stone workers. |
|  | Government aid for TPT | Seeking governmental support and financial aid for TPT procurement, fostering collaboration to address funding challenges. |
|  | Corporate aid for TPT | Exploring support from corporate entities to contribute resources and aid for TPT procurement, diversifying funding sources. |
|  | Rate contract for TPT | Establishing rate contracts for TPT procurement, streamlining the procurement process and ensuring cost-effectiveness. |
| Program capacity building | TPT program capacity building | Crafting comprehensive training programs tailored for healthcare professionals to enhance their proficiency in addressing latent tuberculosis infection and administering tuberculosis preventive treatment regimens effectively. |
|  | Staff training on ADRs | Providing specialized training for healthcare staff on recognizing and managing adverse drug reactions (ADRs) associated with TPT, improving patient care. |
|  | Capacity building for counselling | Strengthening the capacity of healthcare personnel in providing effective counselling services, promoting informed decision-making among agate-stone workers. |
|  | Capacity building for IGRA | Building the capacity of healthcare professionals in conducting and interpreting Interferon-Gamma Release Assay (IGRA) test results for effective diagnosis. |
| Artificial intelligence in occupational health | AI for early silicosis detection | Integrating artificial intelligence (AI) tools for early detection of silicosis among agate-stone workers, enabling proactive health interventions. |
|  | AI for TB-silicosis differentiation | Developing and leveraging AI algorithms to distinguish between TB and silicosis, ensuring accurate diagnosis and appropriate treatment. |
|  | AI algorithm for TPT eligibility | Developing AI algorithms to assess eligibility for TB preventive treatment (TPT), optimizing decision-making and resource allocation. |
