# Supplementary material for: Mixed methods study on latent tuberculosis among agate stone workers and advocacy for testing silica dust exposed individuals in India
Source: Sci Rep. 2024 Jun 15;14:13830. doi: 10.1038/s41598-024-64837-4 (PMC11180111; doi:10.1038/s41598-024-64837-4)
Supplement: Supplementary file 7 — Supplementary Information 7. [file 41598_2024_64837_MOESM7_ESM.docx]

Supplementary Table S5: Co-linearity statistics for variables entered in the multivariable logistic regression model for predicting latent TB infection among agate-stone workers in Khambhat (n=438)

| **Variables** | **Unstandardized Coefficients** | | **Standardized Coefficients** | **t-statistic** | **p-value** | **Co-linearity Statistics** | |
| --- | --- | --- | --- | --- | --- | --- | --- |
|  | **B** | **Std. Error** | **Beta** |  |  | **Tolerance** | **VIF** |
| (Constant) | 0.235 | .085 | - | 2.771 | 0.006 | - | - |
| Urban (vs. rural) residence | .089 | .047 | .090 | 1.900 | .058 | .980 | 1.021 |
| Overcrowding | .077 | .053 | .069 | 1.445 | .149 | .971 | 1.030 |
| Work in silica dust-exposed settings (five-year unit increase) | .027 | .011 | .115 | 2.409 | .016 | .969 | 1.032 |
| Primary work setting vis-à-vis agate stones (polishing-chipping vs. drilling) | .157 | .055 | .140 | 2.849 | .005 | .907 | 1.103 |
| Vaccinated with BCG | .134 | .050 | .127 | 2.645 | .008 | .946 | 1.057 |

VIF: variance inflation factor; TB: tuberculosis; BCG: Bacillus Calmette-Guérin
